# MRI at low field: A review of software solutions for improving SNR

**DOI:** 10.1002/nbm.5268

**Published:** 2024-10-07

**Authors:** Reina Ayde, Marc Vornehm, Yujiao Zhao, Florian Knoll, Ed X. Wu, Mathieu Sarracanie

**Affiliations:** ^1^ Center for Adaptable MRI Technology, Institute of Medical Sciences, School of Medicine & Nutrition University of Aberdeen Aberdeen UK; ^2^ Department of Artificial Intelligence in Biomedical Engineering Friedrich‐Alexander‐Universität Erlangen‐Nürnberg Erlangen Germany; ^3^ Department of Electrical and Electronic Engineering University of Hong Kong Hong Kong China

**Keywords:** deep learning, electromagnetic interference cancellation, image enhancement, *k*‐space sampling, low signal‐to‐noise ratio (SNR), low‐field MRI

## Abstract

Low magnetic field magnetic resonance imaging (MRI) ($B_{0}$ < 1 T) is regaining interest in the magnetic resonance (MR) community as a complementary, more flexible, and cost‐effective approach to MRI diagnosis. Yet, the impaired signal‐to‐noise ratio (SNR) per square root of time, or SNR efficiency, leading in turn to prolonged acquisition times, still challenges its relevance at the clinical level. To address this, researchers investigate various hardware and software solutions to improve SNR efficiency at low field, including the leveraging of latest advances in computing hardware. However, there may not be a single recipe for improving SNR at low field, and it is key to embrace the challenges and limitations of each proposed solution. In other words, suitable solutions depend on the final objective or application envisioned for a low‐field scanner and, more importantly, on the characteristics of a specific low $B_{0}$ field. In this review, we aim to provide an overview on software solutions to improve SNR efficiency at low field. First, we cover techniques for efficient *k*‐space sampling and reconstruction. Then, we present post‐acquisition techniques that enhance MR images such as denoising and super‐resolution. In addition, we summarize recently introduced electromagnetic interference cancellation approaches showing great promises when operating in shielding‐free environments. Finally, we discuss the advantages and limitations of these approaches that could provide directions for future applications.

## Graphical abstract

The re‐emerging interest we see in low‐field MRI brings signal sensitivity and imaging performance at the core of current research. An overview of software strategies to enhance signal‐to‐noise ratio per unit time at low field (*B*_0_ < 1 T) is provided that covers *k*‐space sampling techniques, reconstruction, post‐processing, and electromagnetic interference cancellation.

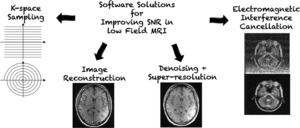

**Abbreviation**
BM*x*D: block matching and *x*D filter
bSSFP: balanced steady‐state free precession
CNN: convolutional neural network
CPU: central processing units
EMI: electromagnetic interference
EPI: echo planar imaging
FSE: fast spin echo
GPU: graphical processing units.
HOSVD: higher order singular value decomposition
MR: magnetic resonance
MRI: magnetic resonance imaging
NMR: nuclear magnetic resonance
PC: personal computer
RAM: random access memory
RF: radio frequency
SNR: signal‐to‐noise ratio
SSDU: self‐supervised learning via data undersampling
TE: echo time
TR: repetition time
VST: variance stabilization transformation

## 1 INTRODUCTION

Since its introduction in the early 1970s, magnetic resonance imaging (MRI) has revolutionized radiology and played a major role in medical diagnosis. Yet, MRI is often perceived as the “last choice” imaging modality because of its high cost (~1 M$ per 1 T) and challenging infrastructure requirements (e.g., complex cryogenic cooling systems, large siting and the RF shielded facilities) limiting therefore its accessibility.^1^, ^2^

Recently, improved dissemination and access to MRI have become a growing paradigm in the MR community.^1^, ^3^, ^4^, ^5^ A straightforward approach is reducing MR scanner cost that derives mainly (but not exclusively) from expensive superconducting magnets used to generate high and homogenous static magnetic fields $B_{0}$ (conventionally: 1.5 or 3 T). This can be made possible working at lower magnetic field strength using simple, cost‐effective electromagnets or permanent magnets, hence releasing MRI scanners from exclusive superconductors and associated needs (e.g., cryogenics, quench systems). Alternatively, mid‐field magnets start to be offered with cryogen‐free magnets, however reducing costs to a smaller extent through lower overall operating costs. Imaging at low $B_{0}$ proves interesting not only cost‐wise but also application‐wise. Compared to high‐field, low‐field MR images are less affected by susceptibility artifacts arising at various interfaces, allowing imaging of regions such as the lungs (i.e., air‐tissue interface) and near implants.^6^ ^,^ ^7^ ^,^ ^8^ The deposited energy in tissue from radiofrequency pulses is lowered which makes MR scanning safer.^9^ In addition, $T_{1}$ relaxation exhibits higher dispersion at low field which could potentially help identifying some pathologies.^10^, ^11^, ^12^ Pushing further accessibility and mobility aspects, low‐field MRI scanners are envisioned as point‐of‐care, lightweight, and shielding‐free devices allowing response to time‐critical clinical questions outside radiology departments.^4^, ^13^

However, recurring concerns are raised about low‐field MRI on achievable performance, mainly tied to the inherently degraded nuclear magnetic resonance (NMR) sensitivity. The raw NMR signal is directly proportional to spin polarization, scaling itself linearly with the magnetic field intensity $B_{0}$. The measured electromotive force induced in a radio frequency (RF) detector is itself proportional to $B_{0}^{2}.$ ^14^ Yet, as for any detection process, performance of the receiver chain in MRI is also affected by noise which generally originates from the coil resistance, preamplifier, and dielectric and inductive losses in the sample (i.e., patient body).^15^ In shielding‐free settings (i.e., without a Faraday cage) envisioned for future point‐of‐care scanners, electromagnetic interference (EMI) is an additional disturbance causing narrow or broadband noise‐like artifacts to superimpose on top of the image's noise floor.

Signal‐to‐noise ratio (SNR) usually is the standard metric used to assess sensitivity. Assuming a fixed noise floor in the MRI detection chain, the overall SNR will consequently drop when measuring at lower $B_{0}$, resulting in noisier signals. When a conventional inductive coil is used as a receiver and considering a quasi‐static electromagnetic analysis (e.g., near field), studies show that SNR scales in the range $\propto B_{0}$ to $B_{0}^{\frac{7}{4}}$ depending on the frequency and the corresponding noise regime.^15^, ^16^, ^17^ At a fixed $B_{0}$, considering a basic gradient‐echo or spin‐echo sequence, the SNR in MR images generally scales with imaging parameters following the equation below^18^:

(1) $$\mathrm{SNR}\propto ∆x∆y∆z\sqrt{N_{\mathrm{avg}}N_{\text{phase}}T_{\text{sampling}}}$$

where the product of the three spatial dimensions, ∆x∆y∆z, represents the voxel volume, $\;N_{\text{phase}}$ is the number of encoding phases, $N_{\mathrm{avg}}$ is the number of signal averages, and $T_{\text{sampling}}$ is the time during which the data acquisition window is open. Equation (1) holds the notorious trade‐off between SNR, resolution, and scan time, inherent to any MRI acquisition. For instance, higher SNR can be reached by averaging repeated acquisitions (increasing $N_{\mathrm{avg}}$), though it increases the total scan time $T_{\mathrm{tot}}=N_{\mathrm{avg}}N_{\text{phase}}T_{R}$, where $T_{R}$ is the repetition time. Unfortunately, prolonged scan times may lead to patient discomfort and increased motion artifacts. Consequently, the ultimate challenge for any MRI acquisition is improving the time‐normalized SNR known as SNR efficiency or imaging efficiency. Normalization to time is done on a per square root of time basis ($\mathrm{SNR}/\sqrt{T_{\mathrm{tot}}}$), leading to the SNR efficiency definition below, for a fixed TR, given by^18^, ^19^:

(2) $${\mathrm{SNR}}_{\text{efficiency}}\propto ∆x∆y∆z\sqrt{T_{\text{sampling}}}$$

Being a common concern for all field strengths, tremendous progress was made in all aspects of MRI, over the past 40 years, to improve the SNR efficiency. Hardware and software solutions have continuously been developed, for the most part at clinical field strengths (i.e., at 1.5 T and above), to enhance the SNR and to reduce scan time. Regarding hardware, significant advances were made on magnet designs, gradients coils, power electronics, and RF instrumentation.^20^, ^21^ As for software aspects, various solutions have been proposed to improve SNR efficiency through prospective or retrospective operations (i.e., applied before acquisition or on acquired data).

Prospective operations involve modifying *k‐*space trajectories and pulse sequence parameters, whereas retrospective operations are leveraged for improving SNR a posteriori through image processing such as denoising and super‐resolution. This latter defies Equation (1) by enhancing terms of the equation (respectively SNR and resolution) without compromising the others. Lately, the emergence of new‐generation graphics cards and access to large databases opened the door to advanced image processing approaches such as deep learning.

Altogether, low‐field regimes may benefit from existing developments but their transfer from high to low fields is not necessarily straightforward. The proposed review aims to give a balanced view on current software developments (i.e., acquisition, reconstruction and post‐processing strategies) for improving SNR efficiency in MRI scanners below 1 T without covering pre‐ or hyper‐polarization.

First, *k‐*space sampling strategies including non‐Cartesian sampling and pulse sequences will be described (**section** 2). Following this, undersampling and image reconstruction techniques will be adderessed (**section** 3). Image processing methods including denoising and super resolution (or image enhancement) will then be covered (**section** 4) before expanding the scope of our paper to recently proposed hybrid (hardware + software) active EMI cancellation in shielding‐free settings (i.e., without a Faraday cage), envisioned for future point‐of‐care scanners, focusing on its software components (**section** 5).

As low‐field imaging is a wide magnetic field range comprising different regimes, each presenting different characteristics and challenges, we will adopt along the paper the following nomenclature inspired by reference^22^: ultra‐low‐field [0, 0.01 T], very‐low‐field (0.01, 0.1 T], and mid‐field (0.1 T, 1 T].

## 2 DATA ACQUISITION STRATEGIES

Acquisition strategies greatly influence image SNR, contrast, and sensitivity to certain artifacts. At low field, SNR is greatly impeded from lower NMR sensitivity and the choice of data acquisition strategy is crucial to provide clinically acceptable acquisition times and relevant images for diagnosis. To date, several strategies have been investigated to increase SNR efficiency, some of which benefit from the specificities of this regime.

### 2.1 K‐space sampling trajectories

Cartesian sampling is the most commonly used trajectory, where each line of *k‐*space is sequentially acquired on a Cartesian grid after a separate RF excitation pulse. Its widespread use is mainly due to its lower sensitivity to imperfections (i.e., off‐resonance, eddy currents) and straightforward implementation. However, Cartesian trajectories may result in long scan times needing to cover all *k*‐space lines on the grid with full readouts, eventually affecting SNR efficiency. According to Equation (2), for a given $T_{R}$, SNR efficiency can be improved by increasing $T_{\text{sampling}}$ or the sampling duty cycle (${\mathrm{SNR}}_{\text{efficiency}}\propto \sqrt{T_{\text{sampling}}}$). Therefore, various sampling trajectories have been proposed for more time‐efficient *k*‐space coverage. Echo planar imaging (EPI) and spiral trajectories are two common examples of fast and SNR‐efficient acquisitions. Despite being SNR‐efficient, these trajectories generally require high receiver bandwidths which can limit the potential gain in SNR. Yet, transverse relaxation $T_{2}$ increases at lower fields^23^, ^24^, ^25^ . Furthermore, absolute homogeneity of the main magnetic field is generally improved when sizeable superconductive magnets or resistive electro‐magnets are used (as opposed to permanent magnet designs), leading to reduced off‐resonance effects while increased transverse relaxation $T_{2}^{*}$ approaches $T_{2}.$ ^8^ Longer $T_{2}^{*}$ and $T_{2}$ hence allow using EPI and spiral trajectories with lower bandwidth (lower noise), thus mitigating the penalty on SNR. Additionally, blurring artifacts caused by $T_{2}^{*}$ decay during readouts in EPI and spiral trajectories may be reduced.

Using simulations, phantom, and in vivo imaging, Restivo et al.^26^ demonstrated the favorable physical conditions of lower fields for long readout acquisitions. Specifically, Cartesian, EPI, and spiral in‐out balanced steady‐state free precession (bSSFP) sequences were compared at 0.55 T for cardiac cine imaging. For the same acquisition time, the SNR in myocardium increased by 45% when using spiral in‐out and by 26% when using EPI compared to Cartesian imaging in Bloch simulations. Additionally, spiral in‐out provided better image quality with limited artifacts related to flow and motion.

**FIGURE 1:**
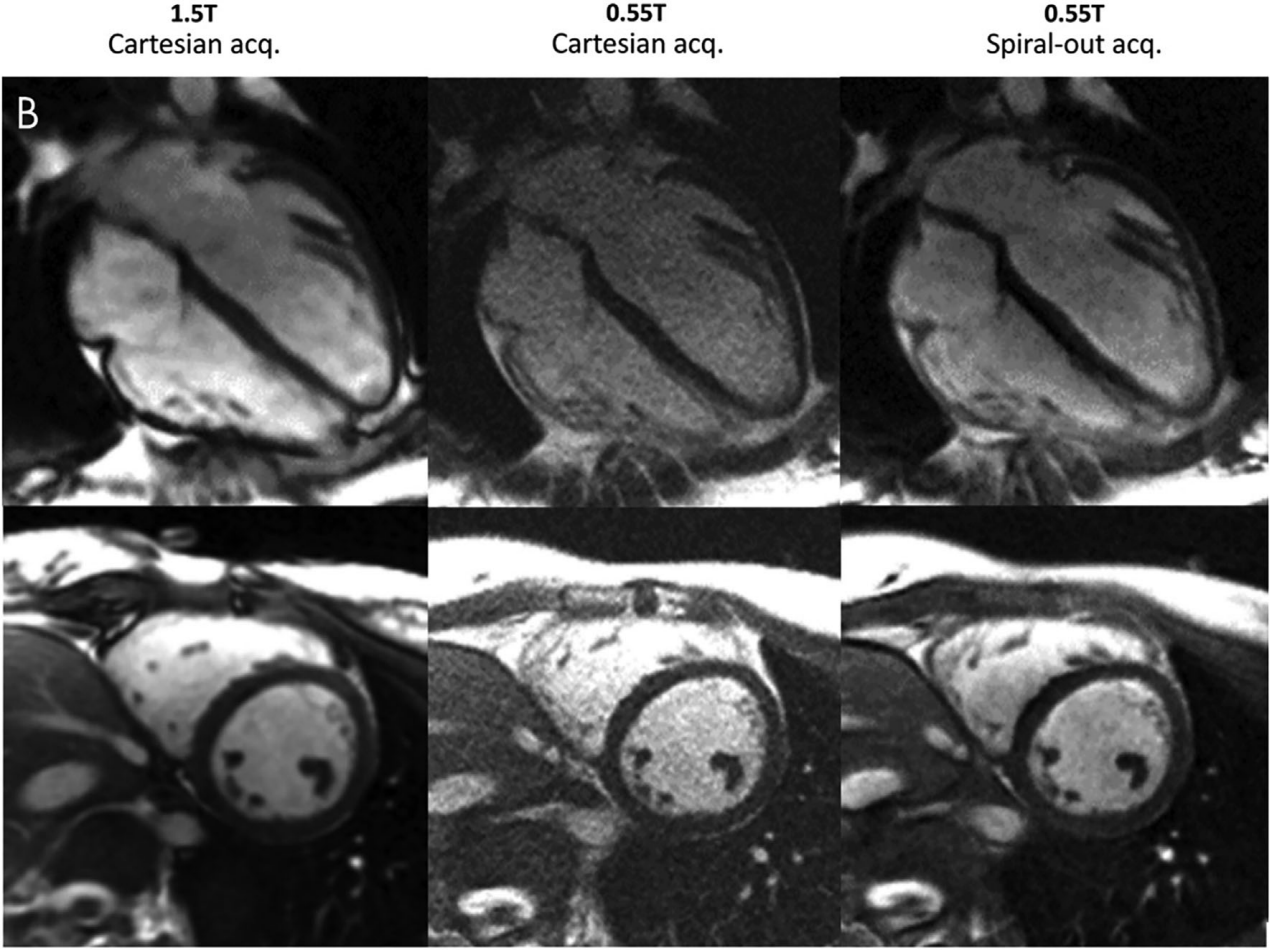
Image reused from Campbell‐Washburn et al.^23^ Balanced steady‐state free precession cardiac imaging demonstrated in a 23‐year‐old woman. Total acquisition time was fixed, but sampling efficiency was increased with spiral acquisitions. Images were acquired with Cartesian sampling at 1.5 T and 0.55 T, and spiral‐out at 0.55 T. Good image quality and SNR improvements at 0.55 T are observed with the spiral sampling scheme. Blood: SNR Cartesian 0.55 T: 15 ± 3; SNR spiral 0.55 T: 28 ± 8, SNR Cartesian 1.5 T: 63 ± 8; myocardium: SNR Cartesian 0.55 T: 6 ± 1, SNR spiral 0.55 T:10 ± 3, SNR Cartesian1.5 T:17 ± 1. On average, SNR recovered to 57% of that measured at 1.5 T. Details of used sequence parameters can be found in the corresponding reference.

Similarly, Campbell‐Washburn et al.^8^ demonstrated considerable improvement in SNR in neuro‐ and cardiac imaging by using spiral compared to Cartesian readouts, while keeping the total image acquisition time the same in both scans (Figure 1). The acquired images showed reduced distortions caused by off‐resonance effects in high susceptibility regions when compared to conventional field strengths. A stack‐of‐spirals trajectory has also been proposed by the same author for 3D whole‐brain MR fingerprinting at 0.55 T.^27^

Like spiral, rosette trajectories are another class of fast *k‐*space sampling leveraging longer signal readouts achievable at lower fields. Rosette trajectories were successfully employed at 0.55 T for MR fingerprinting.^28^, ^29^

Spiral and rosette trajectories have so far mainly been demonstrated on 0.55 T clinical systems with relatively high hardware performance. Mickevicius et al.,^30^ for instance, implemented an MR fingerprinting sequence for MR‐guided radiation therapy and specifically chose radial over spiral sampling due to insufficient gradient strength on their 0.35 T system.

Indeed, fast acquisition trajectories usually require gradients with relatively high performance in terms of slew rates, duty cycle, eddy currents, and accuracy. However, very low‐, ultra‐low‐field devices and point‐of‐care units generally have inferior gradient performance by design compared to most commercial high‐field systems, aiming at improved access and financial value. Consequently, *k‐*space trajectories with long readout durations may be challenging on these devices with potentially increased blurring due to off‐resonance effects. Very‐low and ultra‐low‐field systems in particular may be equipped with permanent instead of superconductive or electro‐magnets, therefore also suffering from temporally and spatially less stable fields compared to superconductive high‐field magnet systems.^20^ Hence, spiral or rosette adoption is an open research topic on such systems.

### 2.2 Pulse sequences

Improved SNR efficiency can be achieved through different pulse sequences. Fast (or turbo) spin echo (FSE), also named rapid acquisition and relaxation enhancement (RARE), enables faster data acquisition while being more resilient to off‐resonance effects. Scan time is reduced proportionally to the echo train length (ETL) when compared to conventional single spin echo sequence with identical parameters.^31^ Consequently, FSE could be an alternative on systems with high $B_{0}$ inhomogeneity such as permanent magnet scanners. Cooley et al used an FSE sequence on an 80‐mT permanent magnet scanner with a built‐in read‐out gradient. The permanent read‐out gradient causes a rapid loss of transverse magnetization (very short $T_{2}^{*}$). Thanks to FSE, the transverse magnetization is refocused with $180^{\circ }$ pulses enabling to efficiently probe the inherently longer $T_{2}$ s at low field.^32^

At higher field strengths, the echo train length of a FSE sequence is limited due to high specific absorption rate (SAR) stemming from the refocusing radiofrequency pulse train. This limitation is reduced at low‐field strengths due to quadratic dependence of SAR with the RF frequency and consequently $B_{0}.$ ^31^ SAR simulations done by Van Speybroeck et al. on a 50‐mT scanner showed that a very long echo train (>128) turbo spin echo with short inter‐pulse times (5–10 ms) can be run within SAR limits allowing efficient data acquisition.^33^ As one of the most frequently utilized pulse sequences in clinical settings, FSE has been employed in numerous low‐field MRI studies spanning various low‐field $B_{0}$ field strengths.^34^, ^35^, ^36^, ^37^

The SAR advantage was also leveraged to exploit a larger range of flip angles at low field. For instance, Liu et al. were able to use higher flip angles without SAR issues to compensate for SNR loss in an MR fingerprinting sequence on a 0.55 T scanner.^29^ Similarly, Sarracanie et al.^38^ optimized acquisition parameters for MR fingerprinting at 0.1 T, including higher flip angles to achieve maximal discrimination and SNR.

In addition to longer $T_{2}$ encountered at lower fields, $T_{1}$ is shortened allowing pulse sequences with reduced TRs and thus reduced acquisition times while maintaining SNR.^10^ Together, the prolonged signal readouts (due to longer $T_{2}^{*}$ and $T_{2}$) and the shortened TR can be used to increase the sampling efficiency and hence the SNR efficiency.

**FIGURE 2:**
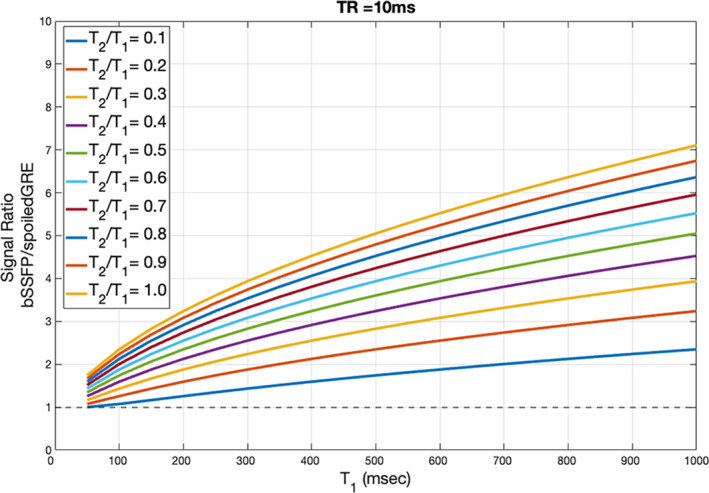
Ratio of bSSFP signal over RF spoiled gradient echo for different $T_{1}$ values, $T_{2}$/$T_{1}$, using optimal flip angles, TR = 10 ms simulating the effect of limited gradients performance, TE = 0 ms. A high signal gain over RF spoiled gradient echo can be achieved, particularly for $T_{2}$/$T_{1}$ ratios close to 1.

One of the most SNR efficient sequences is bSSFP.^39^ This sequence generates a steady state not only for the longitudinal magnetization but also for the transverse magnetization, resulting in a relatively high signal (up to 50% of longitudinal magnetization). The expression of bSSFP signals (with RF phase sign alternation) is given by^31^:

(3) $$S_{\text{bSSFP}}=M_{0}\sin\Theta \frac{1-E_{1}}{1-\left(E_{1}-E_{2}\right)\cos\Theta -E_{1}E_{2}}e^{-\mathrm{TE}/T_{2}}$$

where $M_{0}$ is the proton density, $E_{1,2}=e^{-\mathrm{TR}/T_{1,2}}$. When $\mathrm{TR}\ll T_{1}$ and $T_{2}$, and the angle yielding the highest signal is selected, the signal amplitude reduces to $\;\frac{1}{2}M_{0}\sqrt{T_{2}/T_{1}\;}$. As $T_{2}$‐to‐$T_{1}$ ratio approaches one at decreasing field strengths, bSSFP becomes a promising choice at low field. A Bloch equation–based simulation comparing the signal amplitude ratio of bSSFP to an RF spoiled gradient echo sequence with limited gradient performance is presented in Figure 2. A high signal gain over RF spoiled gradient echo can be achieved, particularly for $T_{2}/T_{1}$ ratios close to 1. Consequently, bSSFP has been used in multiple studies.^8^, ^40^, ^41^, ^42^, ^43^ ^,^ ^44^ Nevertheless, bSSFP is known to have strict requirements on absolute field homogeneity to avoid banding artifacts. Maximum allowed off‐resonance for “banding‐free” images is inversely proportional to TR,^39^ hence setting hard requirements on gradient hardware. At low field though, bSSFP tolerance to off‐resonance effects naturally increases from improved absolute homogeneity.

## 3 IMAGE RECONSTRUCTION

Reconstructing MR images is conventionally done by applying an inverse Fourier transform to full Cartesian *k‐*space. However, in several cases, commonly encountered at low field such as $B_{0}$ field inhomogeneity, non‐Cartesian sampling, or undersampling to accelerate the acquisition, typical Fourier reconstruction is not optimal. In this section, we will review commonly used MR image reconstruction strategies in the context of low‐field imaging, and for general background on MR image reconstruction, we shall refer the reader to references.^45^, ^46^ Before delving into reconstruction methods encountered at low field, it is important to note that, as such methods tend to result in oversmoothed rather than noisy images, SNR in its strict mathematical sense is not a suitable metric for image quality assessment and hence image fidelity metrics compared to a fully sampled ground‐truth image are usually considered.^47^

### 3.1 Analytical methods

When Fourier transform is not applicable, MR image reconstruction can be written as a regularized optimization problem where the goal is to find the $\hat{\vec{x}}$ that minimizes the following function:

(4) $$\hat{\vec{x}}={\text{argmin}}_{\vec{x}}{\left\|\vec{y}-A\vec{x}\right\|}_{2}^{2}+\lambda R\left(\vec{x}\right)$$

$\vec{y}$ is the measurement data in *k‐*space, $\vec{x}$ is the image to be reconstructed, and A is the forward system matrix incorporating coil sensitivities, the Fourier transform, and information about the sampling trajectory. $R\left(\vec{x}\right)$ is the regularization term that represents the a priori knowledge about the reconstructed image weighted by a regularization parameter λ. This will lead to a favorable solution with respect to the regularization term. For instance, it can be used to penalize noisy solutions, hence improving the apparent signal‐to‐noise ratio of the reconstruction. Commonly used regularizers that achieve this, namely Tikhonov and total variation, have been applied in low‐field imaging. The Tikhonov regularizer was used by Hsu et al. to reconstruct undersampled data in radial‐like trajectory acquired on a rotary ultra‐low‐field scanner (50 μ T).^48^ Total variation regularization was used on a 50‐mT permanent magnet aiming at not only increasing SNR but also correcting $B_{0}$ inhomogeneity and gradient non‐linearities.^49^, ^50^ In general, the choice of the regularization function does not depend on the field strength. The optimal regularization weight λ, on the contrary, depends on the noise level in the measurement and hence on the field strength. In the following, we will elaborate on the two main constrained reconstruction approaches that were employed at low field for reconstructing accelerated acquisitions.

#### 3.1.1 Compressed sensing

Compressed sensing^51^ is a well‐established constrained method for reconstructing undersampled MRI acquisitions. Undersampling reduces scan time by collecting fewer measurements in *k*‐space, but it also brings challenges for image reconstruction from its inherent ill‐posedness and lower SNR due to reduced $N_{\text{phase}}$ (Equation 1). Compressed sensing alleviates those challenges by relying on the sparsity of the desired image in a transform domain (e.g., the wavelet transform) in combination with a pseudo‐random sampling. The constrained problem is given by:

(5) $$\hat{\vec{x}}={\text{argmin}}_{\vec{x}}{\left\|\vec{y}-A\vec{x}\right\|}_{2}^{2}+\lambda {\left\|\Psi \vec{x}\right\|}_{1}$$

where Ψ transforms the image into a sparse domain (such as the wavelet domain). Compressed sensing relies, however, on a sufficiently high baseline SNR which may not be available at low‐field strength. More specifically, compressed sensing becomes impractical if random noise levels exceed that of the incoherent undersampling artifacts, as random noise does not have a sparse representation. Simonetti and Ahmad demonstrated (retrospective undersampling) compressed sensing reconstructions using an acceleration rate of two for cardiac MRI at 0.35 T.^43^ They argued that, at low field, compressed sensing may enable moderate acceleration rates of 2 or 3 while benefiting from less structured artifacts caused by field inhomogeneity when compared to high field counterparts. Varghese et al. demonstrated an entire cardiovascular MRI examination on a clinical 0.55 T scanner with acceleration rates between three and 13 depending on the acquisition specifications.^52^ The authors note, however, that stronger regularization was required, leading to patch‐like artifacts in their reconstruction results. Tamada et al.^53^ proposed a compressed sensing reconstruction for a 1‐T permanent magnet using cross‐sampling of *k*‐space to improve incoherence of the undersampling artifacts. Their method further includes a correction of off‐resonance effects due to $B_{0}$ field inhomogeneity and acheived 2.5‐fold acceleration.

Compressed sensing was further successfully used on an ultra‐low‐field 6.5 mT system by Sarracanie et al. for the reconstruction of 3D Overhauser‐enhanced MRI undersampled by a factor of up to 3.5.^41^ Above this acceleration rate, significant artifacts remained. Likewise, an acceleration factor of two was achieved by Koolstra et al. using compressed sensing in combination with a model‐based approach on a 50‐mT permanent magnet system.^50^

#### 3.1.2 Parallel imaging

Parallel imaging is routinely used in MRI to reduce scan time through *k‐*space undersampling. Parallel imaging reconstruction can be equally expressed as an optimization problem with a coil sensitivity‐based constraint. This technique exploits the distinct spatial coil sensitivity information of phased array coils to omit some phase‐encoded steps. However, it is well‐known that acceleration benefits come at the expense of reduced SNR. Specifically, reconstructed images are not only subjected to noise increase due to undersampling but also to non‐uniform noise amplification across the image known as the g‐factor (> 1), due to ill‐conditioning of the image reconstruction process.^54^ The g‐factor depends on the acceleration factor *R* (>= 1) and coil configuration among others. The SNR of an accelerated acquisition with parallel imaging is given by ${\mathrm{SNR}}_{\text{accelerated}}=\frac{{\mathrm{SNR}}_{0}}{g\sqrt{R}}$, where ${\mathrm{SNR}}_{0}$ corresponds to the SNR of an unaccelerated acquisition. Nevertheless, it was shown that, under certain conditions, acquisition time reduction does compensate the SNR loss and leads to an improvement in SNR efficiency.^55^, ^56^

At lower field strengths, few studies in the mid‐field range (0.55 T) have been identified using parallel imaging reconstruction such as Sensitivity Encoding (SENSE)^57^ and Generalized Autocalibrating Partially Parallel Acquisitions (GRAPPA),^58^ with predominantly an acceleration factor of two.^26^, ^52^ Interestingly, only one study at very‐low‐field employed conjugate gradient (CG)‐SENSE on a Hyperfine Swoop scanner (64 mT) (Hyperfine Inc, Guilford, CT, USA, www.hyperfine.io) equipped with eight receiver phased‐array coils achieving an acceleration of 2 and 4.^35^

The limited number of studies using parallel imaging, particularly at very‐low‐ and ultra‐low‐fields, despite the techniques being well‐established in conventional field strengths, may hint at challenges preventing its widespread adoption. Indeed, due to its inherent property to amplify noise, parallel imaging methods are better suited to natively higher SNR regimes where a potential decrease in SNR does not affect diagnostic outcomes.^59^ Furthermore, it is still unclear from the literature if a potential gain in SNR efficiency could be achieved at low field under certain conditions similar to the approach undertaken by Weiger et al.^55^ Ultimately, very‐low‐ and ultra‐low‐field systems are generally equipped with single‐channel detectors or phased arrays comprising fewer and larger elements than high field settings^60^ which results in a higher g‐factor for a given acceleration rate. This primarily arises from the lack of a rationale behind phased‐array in low‐field regimes. Contrary to high field, noise sources at very low field (i.e., < 5 MHz) come predominantly from the coils rather than the sample/patient, which limits the utility of using phased array coils with a large number of elements.^31^, ^61^ Additionally, fewer element phased‐arrays allow to improve cost‐effectiveness, patient comfort, and system portability.

### 3.2 Learning‐based methods

Various learning‐based methods have recently been proposed for MR image reconstruction where the space of the sparse representation is not defined explicitly, as in compressed sensing, but learned from the data.^46^ Such methods do not suffer from noise amplification like parallel imaging. However, they require a certain amount of information (i.e., sufficiently high SNR in the acquired data) to produce high‐quality and reliable results. Again, SNR in its mathematical sense is not a suitable metric for images reconstructed using these methods. Hence, we refer to image quality in a broader sense instead of SNR in the following.

One of the earlier learning‐based methods proposed was dictionary learning, also known as sparse coding.^62^ Ahishakiye et al. built on this approach and proposed a dictionary learning method with adaptive‐size dictionaries for MR image reconstruction particularly for a portable, Halbach array–based low‐field scanner developed for pediatric imaging in low‐ and middle‐income countries.^63^ Their approach includes learning an adaptive‐size dictionary and sparse representations of patches from a zero‐filled reconstruction of undersampled *k‐*space data. The authors demonstrate reconstructions of 2D images retrospectively undersampled using a 2D variable density sampling mask with an undersampling factor of 10. In these reconstructions, however, significant artifacts remain.

**FIGURE 3:**
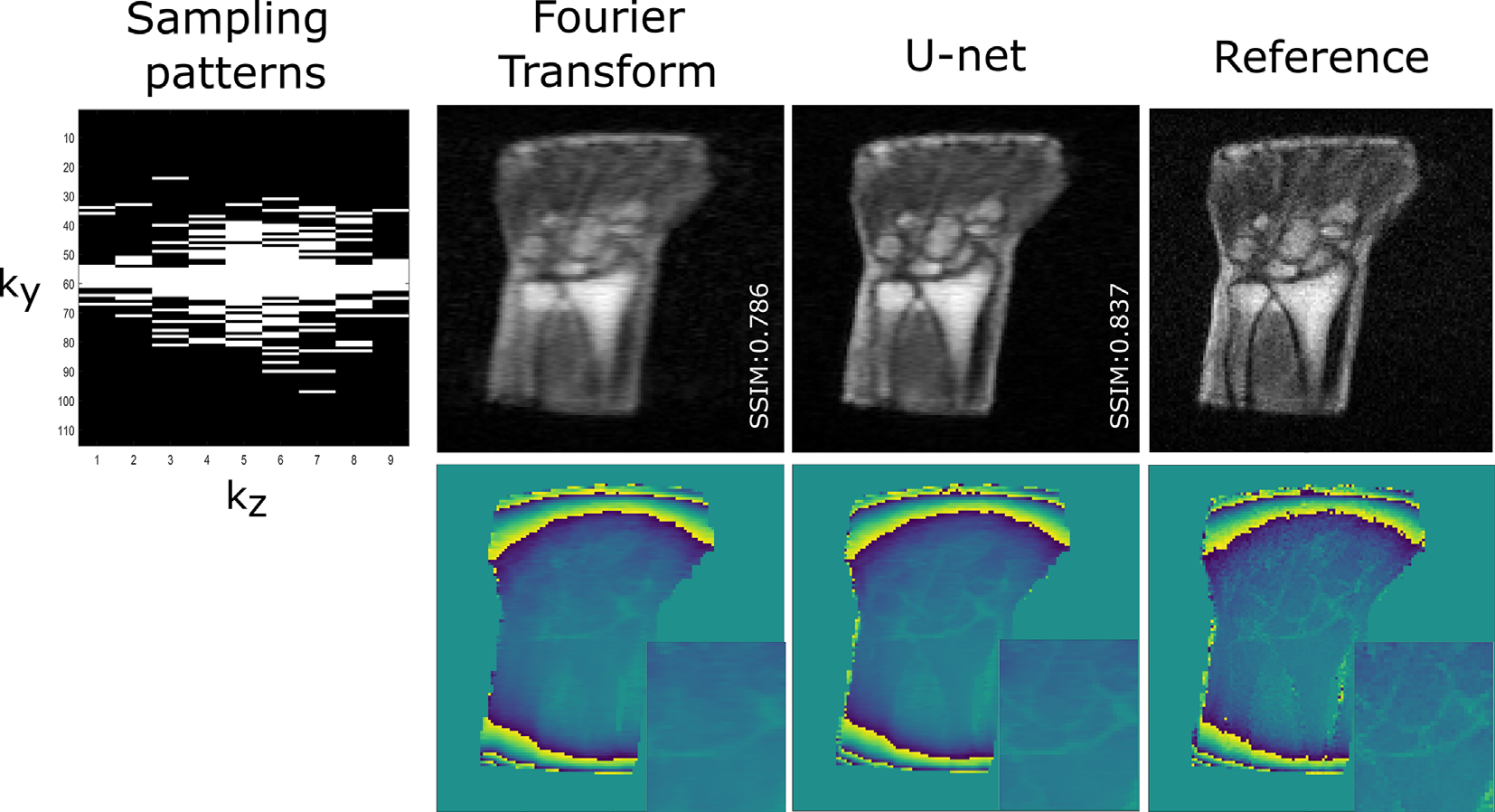
Image modified from Ayde et al.^64^ Magnitude and phase images of a five fold accelerated acquisition (20% sampling) reconstruted with Fourier transform and residual U‐net. The human wrist images were acquired on a 0.1‐T scanner with the following imaging parameters: spoiled gradient echo, voxel size: 1.2 × 1.2 × 6.3 mm^3^, TE/TR 7.2/31 ms, number of averages 28, and fully sampled acquisition time 14 min 56 s.

Another class of learning‐based approaches is deep learning reconstruction. This can be subcategorized depending on the problem formulation. The first category operates purely in image space and tries to remove undersampling artifacts in the image domain. Ayde et al.^64^ proposed an image‐to‐image approach to reconstruct magnitude and phase information of undersampled single‐coil human wrist data acquired at a 0.1 T scanner. Data augmentation was employed to mitigate data scarcity. The authors also studied the effect of random Gaussian sampling patterns with different variances on their algorithm. Interestingly, the sampling pattern with the fewest acquired high‐frequency samples, primarily leading to increased global blurriness, resulted in the best image quality. This implies that the prior knowledge obtained from the limited database helped to correct the global artifacts by improving image sharpness, but was insufficient for recovering fine details (Figure 3).

In a second type of approaches, a direct mapping from *k*‐space to reconstructed image is learned. One method of this kind, called AUTOMAP, has originally been proposed by Zhu et al.^65^ for high‐field data and uses fully connected and convolution layers.

**FIGURE 4:**
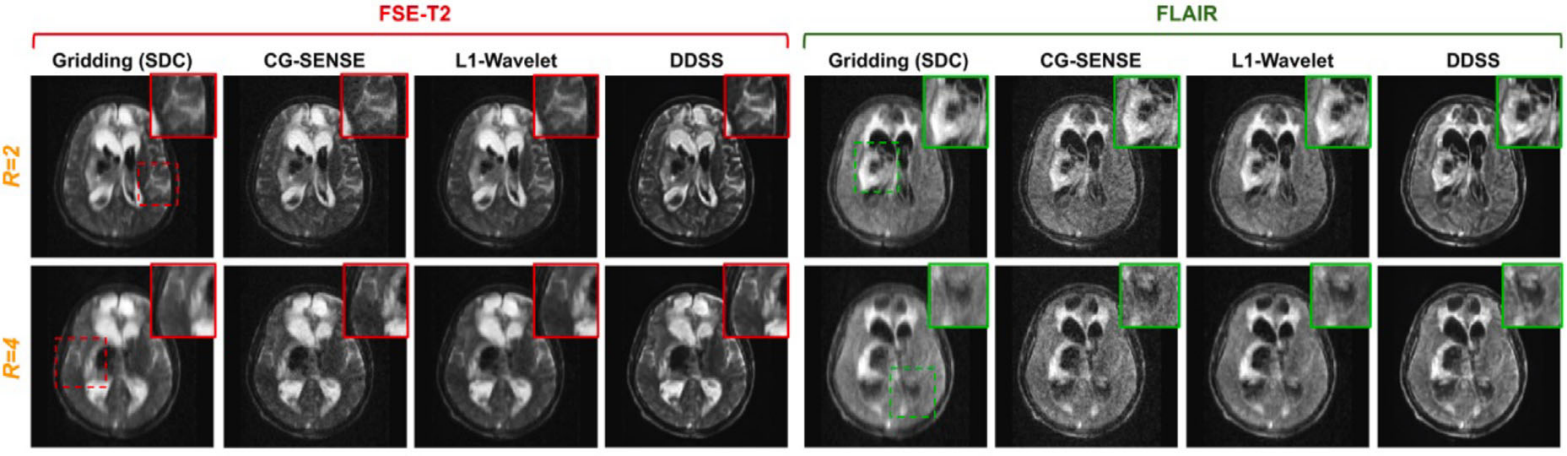
Image reused from Zhou et al.^35^ Qualitative comparisons of FSE‐$T_{2}$ weighted and FLAIR reconstructions from real data acquired from a low‐field (64 mT) MRI scanner. The subject was diagnosed with a hemorrhagic stroke with associated midline shift. The proposed self‐supervised reconstruction (DDSS) is compared to gridding,^67^ L1‐wavelet,^68^ and CG‐SENSE^69^ reconstructions.

A third category of deep learning–based MRI reconstruction methods, known as unrolled or physics‐guided networks, aims to solve the regularized minimization problem described in Equation (4) by “unrolling” it and learning the regularization term $R\left(\vec{x}\right)$ from data. This approach is appealing as it ensures consistency of the reconstruction with the acquired *k‐*space data. Schlemper et al.^66^ and Zhou et al.,^35^ respectively, applied this concept to data acquired at a 64‐mT portable point‐of‐care MR scanner. Schlemper et al. used high‐field data for training of a non‐uniform variational network. High‐field data was corrupted using random jittering of *k‐*space points to simulate effects the authors expected to encounter on their 64 mT scanner, such as eddy currents and patient motion. The network performance was then tested on 3D acquisitions of the human brain prospectively accelerated by a factor of 3.5 using two‐dimensional variable density Poisson disk sampling. Although their method produced less noisy reconstructions than compressed sensing, there seemed to be a systematic bias (visually) towards increased signal intensities in the learnt reconstructions, possibly owed to the training performed on high‐field data. Zhou et al.^35^ later proposed a self‐supervised training scheme for a similar network architecture that can be trained without fully‐sampled reference data. The authors could avoid a distribution mismatch between training and test datasets by training the network directly on real (i.e., acquired) low‐field data. Their method outperformed traditional baseline methods such as gridding, CG‐SENSE, and L1 wavelet reconstructions (Figure 4).

## 4 ADVANCES IN IMAGE PROCESSING

### 4.1 Denoising

In MRI, data are acquired as complex values (*k*‐space) which are corrupted by thermal noise.^15^ This noise is well modeled by a Gaussian distribution with zero mean and variance $\sigma^{2}$ in the real and imaginary parts of the complex data. Image reconstruction is normally done by applying an inverse Fourier transform to the acquired data. Since the Fourier transform is linear, the Gaussian distribution of noise is retained in the complex images. Generally, it is the computed magnitude of these complex images that is used for diagnosis. Such non‐linear magnitude transformation then alters the distribution of the noise from Gaussian to Rician.^70^ Unlike Gaussian noise, Rician noise depends on the local intensity in the image, making signal‐noise separation difficult.^71^ For high SNR, the Rician distribution is well‐approximated by a Gaussian distribution. When SNR < 2, the Rician distribution deviates from the Gaussian distribution and takes a non‐symmetrical shape, converging towards a Rayleigh distribution once the signal becomes zero (background region).^70^

As previously mentioned, the SNR in low‐field MR images is heavily impacted by inherently lower NMR sensitivity. Denoising can therefore be a crucial step to improve SNR towards clinically acceptable scan‐times and clinical relevance. Denoising generally consists of reducing image noise while preserving the imaged object's integrity (e.g., contrast, overall shape and details) and avoiding the introduction of artifacts. Over the past four decades, various image denoising methods were proposed for denoising MR images, some of which have been employed in low‐field settings.

In the following section, we review some of the most common denoising methods that have been used in low‐field MRI initiatives over the past 20 years. To this end, we broadly classify these methods into two categories: (1) analytical methods, comprising spatial domain and transform domain methods, and (2) deep learning–based methods.

#### 4.1.1 Analytical methods

##### Spatial domain methods

Spatial domain filters aim to remove noise by applying the operations directly to the image matrix.^72^ Spatial filters can be sub‐divided into two categories: local and non‐local filters. Local filters exploit the correlation among neighboring pixels. A qualitative study compared the performance of a group of spatial local filters on 0.5 T MR images.^73^ The results showed that bilateral and anisotropic diffusion filters led to better denoising results than median, Gaussian, and Wiener filters. The latter suffer indeed from a non‐context behavior leading to undesirable edge smoothing and loss of details. In contrast, bilateral filters address this problem by applying kernels that depend not only on Euclidean distance between pixels but also on range (or intensity) differences allowing edge preservation. In the same context of edge preservation, anisotropic diffusion^74^ aims at solving a modified heat equation (partial differential equation), where the constant‐diffusion coefficient is replaced by a gradient‐based coefficient. Hence, the diffusion process occurs most preferably in homogenous areas and is minimized on edges. Considering its efficiency in terms of computational complexity, anisotropic diffusion filters have been successfully applied in recent low‐field MRI work.^42^ The main drawback of anisotropic diffusion filters is the occurance of the so‐called “staircase” effect due to transformation of smooth regions into piecewise constants. In addition, local filters' performance generally tends to decrease when the noise level is high because the correlations of neighborhood pixels are highly affected by high noise levels.^75^

Subsequently, methods exploiting non‐local similarities among patterns in the image were developed and proved more robust to noise than local filters.^76^ Non‐local filters have been applied to low SNR high field images. Yet, to the best of our knowledge, no low‐field study solely used non‐local filters.

Mathematically, denoising can further be considered as an optimization problem where the denoised image $\hat{x}$ is obtained by minimizing an objective function (cf. Section 3.1, Equation 4). Based on this approach, de Leeuw den Bouter et al.^49^ proposed an algorithm that can be used not only for denoising but also for data reconstruction in case standard Fourier transform shall fail (i.e., in case the main static magnetic field and spatial encoding gradients are non‐uniform and/or non‐linear). Promising results were achieved on a 50‐mT MR scanner^77^ with total variation adopted as a regularizer. Convergence was reached within ~36 s for a 3D image of 128^3^ pixels performed a desktop PC with an IntelI XI(R) W‐2123 CPU 3.6 GHz.

Working on the same low‐field system and focusing solely on efficient denoiser for low‐resource settings, Shan et al. evaluated different solution methods combined with different priors for the conventional optimization problem. They found that a total variation prior, combined with a suitable solver, gave the best qualitative results in less than 15 s for a 3D image of 128^3^ pixels, carried out on an Apple MacBook Air equipped with an M1 CPU and 16 GB of RAM.^78^

##### Transform domain methods

In contrast to spatial domain filtering methods, transform domain filtering operations are carried out on a basis function that exhibits sparsity (i.e., signal can be represented by fewer number of non‐zero coefficients).^72^ Rosenbaum et al. investigated this approach for MR images acquired at 0.35 T,^79^ by using the wavelet transform along with Nowak's algorithm^71^ to account for Rician noise distribution in magnitude images. In a more recent work, a denoising framework called Deep Resolve Gain exploiting the wavelet domain was introduced to MAGNETOM Free. Max scanners^80^ operating at 0.55 T. Specifically, this framework was developed to address the spatially varying noise distribution occurring mostly in parallel imaging. Noise maps were integrated as an additional information to enable a more effective denoising process.^81^

**FIGURE 5:**
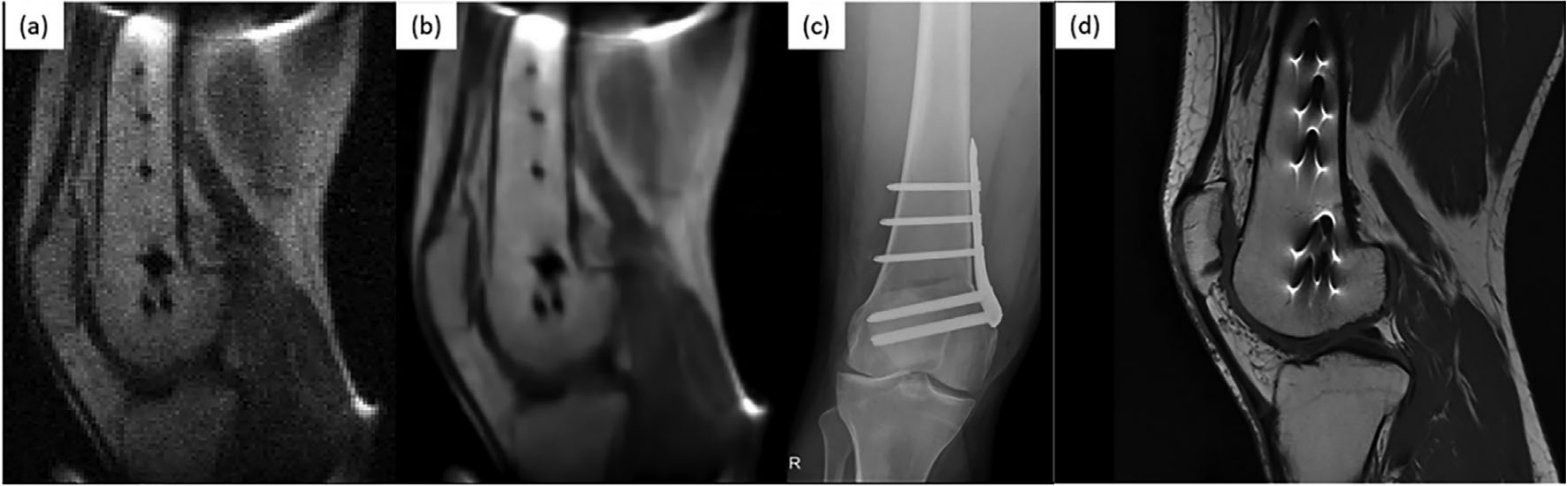
Image modified from Guallart‐Naval et al.^84^ Image of fixation metallic implant attached to the femur, consisting of a plate and seven screws: (A) (9 mm slice from $T_{1}$‐weighted 3D‐RARE acquisition with in‐plane resolution of 1.3 × 2 mm^2^, 12 min scan time, 8 years after femoral shaft osteotomy); (B) same, but BM4D‐filtered and rescaled by ×2 to increase the number of pixels; (C) lateral X‐ray computed radiography (2 weeks after surgery); (D) sagittal view of the same knee, acquired with a Siemens Skyra 3 T system ($T_{1}$‐weighted 2D‐RARE acquisition with slice thickness 3.9 mm and pixel resolution 0.26 × 0.26 mm^2^, 1 year after surgery).

In 2007, block matching and 3D filter (BM3D) was proposed by Dabov et al.^82^ This method combines the advantages of two approaches: (1) the similarity of patterns/patch present in the image known as non‐local filters concept and (2) the transform approaches. In a nutshell, it is based on grouping similar patches into a 3D array and then filtering the latter array using sparse representations in the transform domain. Grouping similar patches has the advantage of enhancing sparsity and is the reason behind the notable performance of BM3D. BM4D presents an extension of BM3D to volumetric data. It was further adapted to MR images with Rician distributed noise by applying, before denoising, variance stabilization transformation (VST) to convert non‐Gaussian noise distribution into standard deviation across the image.^83^ The final output is obtained by inverse VST of the denoised BM4D images. Considered as one of the most powerful denoising methods, BM*x*D was adopted in many low‐field studies not only as a denoiser but also as an evaluation standard for denoisers (Figure 5).^84^, ^85^ ^,^ ^86^

**FIGURE 6:**
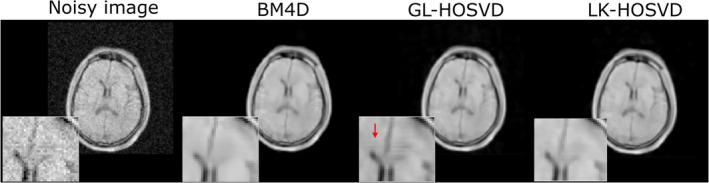
Image modified from Zhang et al.^86^ Different denoising approaches on brain MR data acquired from a 50‐mT scanner with a single average (noisy image) were compared. The results of denoising with BM4D, GL‐HOSVD, and the proposed approach LK‐HOSVD with corresponding enlarged version are shown. Red arrows refer to introduced stripe artifacts.

In 2015, Zhang et al. exploited similarity and sparsity for denoising MR images using higher order singular value decomposition (HOSVD).^87^, ^88^ In contrast to BM*x*D that uses universal bases (or dictionaries) such as wavelet and discrete cosine, the SVD bases are learned from the image (dictionary learning) whose inherent redundancy may lead to a sparser representation than fixed methods. While comparable in computational complexity, HOSVD offers a simpler approach with fewer free parameters to set.^88^ Further, Zhang et al. proposed a pre‐filtering stage on the global image (GL‐HOSVD) to mitigate stripe artifacts with lower‐SNR images (such as diffusion weighted images).^89^ However, experimental results revealed that GL‐HOSVD may still lead to artifacts and blurred details when dealing with higher noise levels such as those encountered at field strength lower than 0.1 T. In 2022, a new version of the HOSVD denoiser was proposed, termed LK‐HOSVD, with a pre‐filtering step applied in the *k*‐space domain.^86^ Results indicated better denoising performance with less artifacts for images acquired at 50 mT compared to BM4D and other SVD‐related methods (Figure 6). LK‐HOSVD denoising of a 3D image with matrix size:176 × 128 × 24 took 16 min on a standard laptop with 2.9 GHz CPU and 16 GB RAM.

#### 4.1.2 Deep learning–based methods

Deep learning–based denoisers are gaining attention due to remarkable performance^90^, ^91^, ^92^ and growing access to graphical processing units (GPUs). Despite being more flexible in handling different noise levels,^93^ analytical methods suffer from several drawbacks generally needing manual parameter selection and a time‐consuming “testing” (or denoising process) phase. Deep learning approaches on the contrary are very efficient in the testing phase^92^, ^93^ while most of the computational burden is shifted to the learning phase (training) usually done offline.^94^, ^95^

Several deep learning approaches were recently proposed to improve SNR at low field with promising performance,^96^ sometimes superior to BM4D/BM3D.^85^, ^97^ However, deep learning networks are usually trained with extensive datasets. Public databases for low‐field data are starting to emerge, like the M4Raw dataset^98^ which contains brain image data acquired at 0.3 T. However, these datasets are inherently specific to a certain field strength and system setup and therefore SNR. Indeed, a major challenge at low field is the lack of large‐scale databases acquired under the same magnetic field strength. To overcome this, investigators have relied on the processing of high‐field MR data to emulate their low‐field counterparts, and trained their networks on variable noise levels to provide enhanced robustness to noise (or generalization). Le et al. proposed a processing pipeline to generate training data for deep learning networks from high field databases. This pipeline emulates data produced at 60–67 mT (Promaxo, Oakland, USA, https://promaxo.com/) by taking high‐field data and adding artifacts caused by undersampling, $B_{0}$ field inhomogeneity, gradient non‐linearity, and noise.^96^

### 4.2 Super‐resolution

Super‐resolution is a post‐processing method that refers to reconstructing an image with higher resolution from either one or multiple distinct low‐resolution images of the same object. At low field, the achievable resolution of MR images is often limited by SNR and total scan time. Hence, super‐resolution could be of interest since it offers the possibility to reconstruct high‐SNR and high‐resolution images without compromising on scan time. In most MRI machines, basic interpolation is done by zero‐padding the *k*‐space data. The Fourier transform of a zero‐padded *k*‐space results in an improved apparent spatial resolution by SINC‐interpolated pixels, but it does not add information nor recover higher spatial frequencies.^31^ In contrast, super‐resolution techniques effectively improve resolution by adding new information, and different techniques were proposed to improve in‐plane or through‐plane resolutions. An overview of super‐resolution methods used in MRI is given in Van Reeth et al.^99^ Like denoising, super‐resolution post‐processing methods can be classified into two main categories: (1) analytical methods and (2) deep learning–based methods.

#### 4.2.1 Analytical methods

**FIGURE 7:**
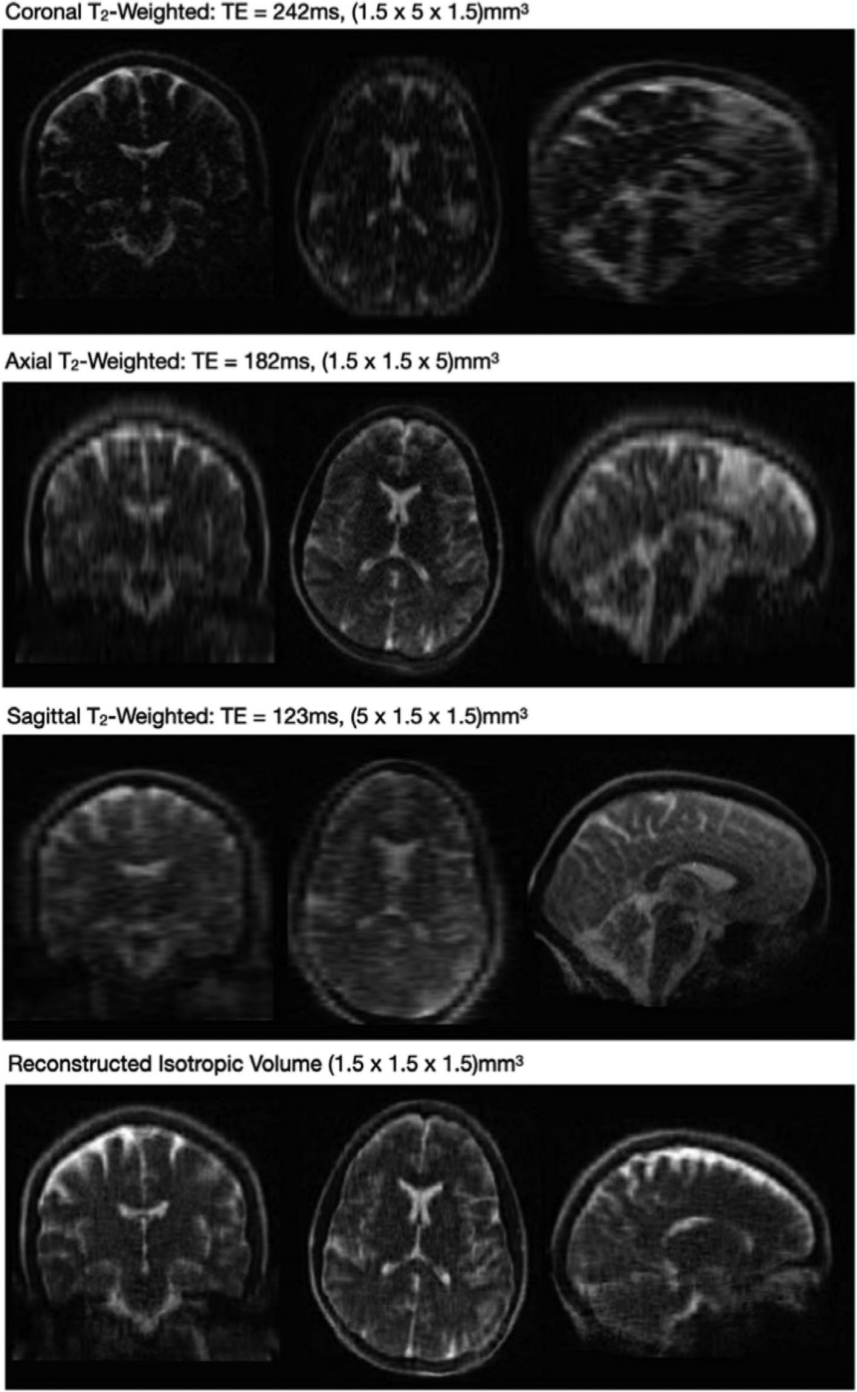
Image modifed from Deoni et al.^102^ Anisotropic $T_{2}$‐weighted data acquired at 64 mT (Hyperfine Swoop scanner) with coronal, axial, and sagittal orientations (top panels); SR reconstruction of an isotropic 1.5 mm $T_{2}$‐weighted image from the acquired data (bottom panel).

Many MRI super‐resolution implementations are based on the combination of lower‐resolution images acquired from different orientations.^100^, ^101^ Often, MRI scans have indeed a fine resolution along two scan directions (i.e., in‐plane resolution) and a lower resolution dimension (generally referred to as the third dimension). Various super‐resolution algorithms can then be used to reconstruct the targeted higher‐resolution image. Deoni et al.^102^ have leveraged this approach for enhancing the resolution of quantitative $T_{2}$ images known to require lengthy acquisition times. The authors managed to reconstruct $T_{2}$‐weighted and quantitative $T_{2}$ images simultaneously with a 1.5‐mm isotropic voxel resolution acquired within 12 min on a Hyperfine Swoop system. This was made possible by combining the information from three acquired anisotropic partitions (1.5 × 1.5 × 5 mm) with orthogonal orientations (i.e., axial, sagittal, and coronal). Each acquisition orientation only has one low‐resolution dimension that is later refined by the acquisition of the other orientations (Figure 7). The same super‐resolution approach was used to investigate the sensitivity of a low‐field scanner to multiple sclerosis lesions.^103^ Three sets of anisotropic orthogonal $T_{1}$‐ and $T_{2}$‐weighted and $T_{2}$‐flair acquisitions were acquired with the Hyperfine Swoop to synthetize higher‐resolution isotropic images.

#### 4.2.2 Deep learning–based methods

Deep learning–based methods also proved suitable for super‐resolution tasks applied to MR images.^104^ ^,^ ^105^, ^106^, ^107^ Siemens Healthineers leveraged deep learning–based super‐resolution in their image processing pipeline in MAGNETOM Free. Max 0.55 T systems (Siemens Healthineers, Erlangen, Germany) using the “Deep Resolve Sharp” framework to enhance MR images with additional information corresponding to the outer parts of *k*‐space. This is essentially done by training a deep convolutional neural network on pairs of low‐ and high‐resolution data.^81^

**FIGURE 8:**
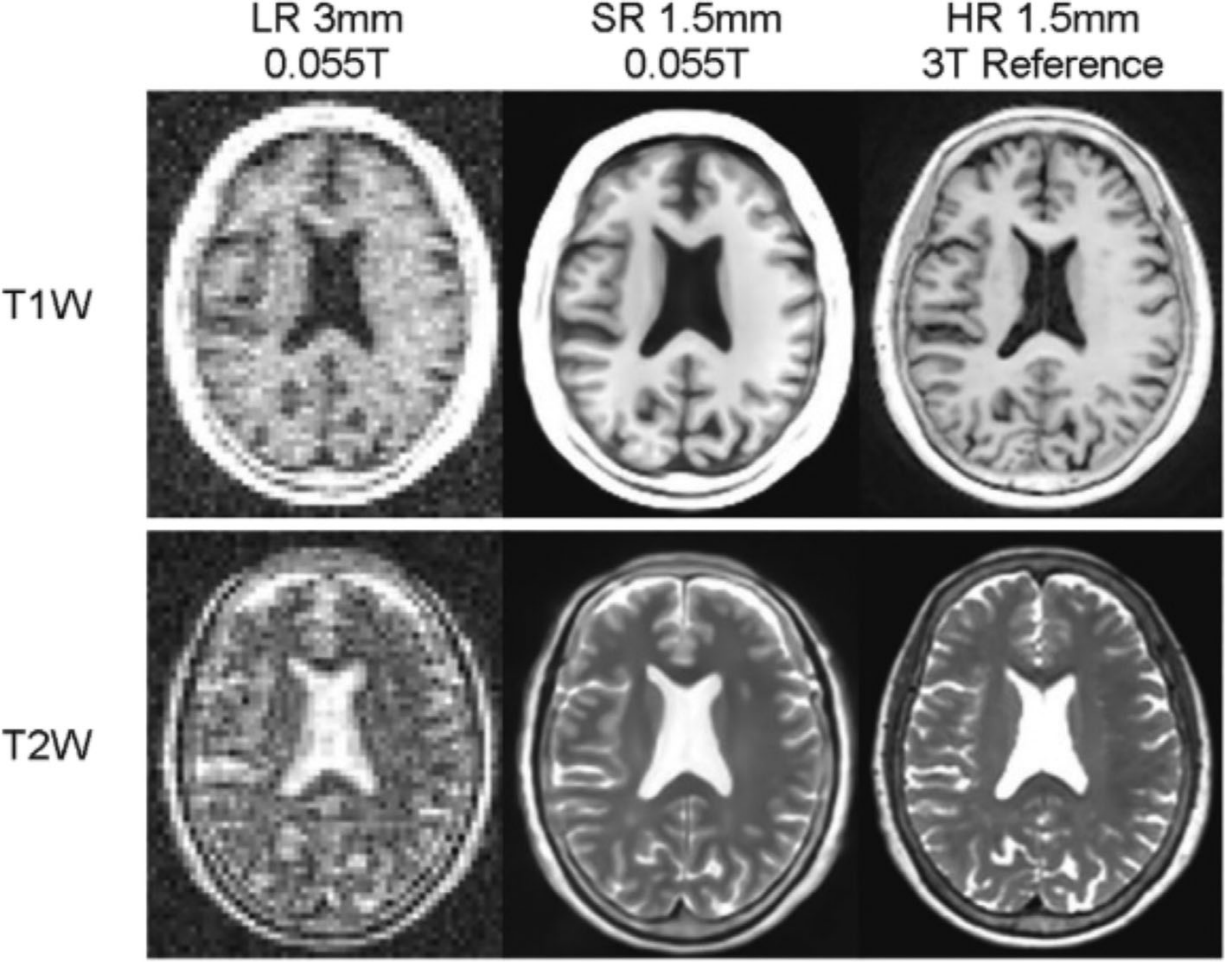
Image reused from Lau et al.^113^ Application of deep learning models to low‐resolution 55 mT MRI datasets acquired from a 67‐year‐old woman. The low‐resolution sets have 3‐mm isotropic resolution. Deep learning super‐resolution output datasets have 1.5‐mm isotropic resolution. $T_{1}$‐weighted ($T_{1}$w) and $T_{2}$‐weighted ($T_{2}w$) axial images are shown, together with the corresponding high‐resolution 3 T images with 1.5‐mm isotropic resolution.

In the same context, de Leeuw den Bouter et al. trained a deep learning network architecture, SRDenseNet,^108^ to enhance in‐plane resolution in 50 mT MR brain images.^109^ They relied on a publicly available high field database, fastMRI,^110^ to train their network. Data from fastMRI were pre‐processed to obtain pairs of noisy data with matrix size: 64 × 64 (input), and noise‐free data with matrix size: 128 × 128 data (output). However, this approach may lead to an undesired contrast transfer from high‐field to low field when reconstructing low‐field data. To mitigate this problem, Iglesias et al. proposed a deep learning–based method, termed LF‐synthSR, to generate high‐resolution isotropic low‐field images. This method is an extension of synthSR^111^ for low‐field data where the training set is generated by a data generator simulating low‐ and high‐resolution images with a wide combination of contrast, orientation, subject motion, bias field, etc. The main goal of the generator is not to faithfully reproduce MR images but to generate a diverse set of input/output images and make the convolutional neural network robust to domain shifts. Iglesias et al. applied this method to provide isotropic resolutions with the Hyperfine Swoop system for using automatic segmentation tools and performing quantitative morphometry.^112^ Due to computational memory limitations, the abovementioned deep learning studies performed learning on 2D space images, with 2D convolutional weights ignoring thus the relationship between adjacent images (3D space). Very recently, Lau et al. proposed a 3D super‐resolution approach to enhance the resolution of 55 mT brain MR images.^113^ The model consists of three modules: the feature extractor, attentional fusion, and reconstruction modules that perform a twofold up‐sampling factor among all three spatial dimensions. Training was done with 3D high‐resolution 3 T MR brain Human Connectome Project data.^114^ Images were downsampled to reach 3 and 1.5 mm isotropic resolution, respectively forming the input and output data of the model. Rician noise was added to the 3‐mm resolution input data to match 55 mT noise levels. Results on healthy volunteers and patients proved promising as significant resolution improvement was demonstrated while attenuating noise and artifacts (Figure 8). More recently, a work from the same group^115^, ^116^ further augmented this model by removing dual acquisition while accomonding partial Fourier sampling. With such partial Fourier super‐resolution reconstruction, 55 mT brain $T_{1}$ W and $T_{2}$ W imaging has been shown to be significantly accelerated.

## 5 ACTIVE EMI SUPPRESSION

Electromagnetic interference (EMI) signals from undesirable RF sources (e.g., nearby electronic devices/equipment, andbroadcasting/communication stations) can contaminate MRI signals. Despite being coherent and not having the statistical characteristics of noise, EMI can drastically impact the perceived SNR in the image space. EMI cancellation is emerging as a crucial reasearch topic for very‐low‐ and ultra‐low‐field systems intended for placement in plural, non‐shielded environments, thereby shifting the paradigm from conventional scanners commonly found in radiology suites.

RF shielding by means of a Faraday cage introduces stringent installation requirements and generally preclude portability and mobility desired in point‐of‐care applications (e.g., in intensive care units, surgical suites). Moreover, such passive RF shielding is inadequate to prevent internal EMI signals that may arise from MRI scanner electronics (e.g., gradient and RF amplifiers, as well as their power supplies).

One strategy to achieve RF shielding‐free MRI is to suppress EMI via active EMI sensing and retrospective cancellation through post‐processing. In essence, for a specific EMI source or environment, the relationship among EMI signals detected by multiple receive coils located at different positions/orientations can be well characterized by the coupling or spectral domain transfer functions among coils.

Basic signal model

To intuitively illustrate how active EMI suppression can be achieved, one can start with a simplified scenario of only two receive coils. They are termed as MRI receive coil and EMI sensing coil, with one detecting both MRI signals and EMI signals, and the other detecting only EMI signals (due to its location). In the absence of noise, one‐dimensional (1D) frequency encoding signals simultaneously acquired by MRI receive coil and EMI sensing coil are modeled as:

(6) $$\begin{array}{c} y_{r}\left(t\right)=s_{r}\left(t\right)+e_{r}\left(t\right)\\ y_{s}\left(t\right)=e_{s}\left(t\right) \end{array}$$

where *s*_*r*_(*t*) and *e*_*r*_(*t*) denote the time domain MRI signal and EMI signal components in MRI receive coil signal *y*_*r*_(*t*), respectively, while *e*_*s*_(*t*) denotes the EMI signal component in EMI sensing coil signal *y*_*s*_(*t*). Assume that only one EMI source exists and it emits EMI signal *i*(*t*), the detected EMI signal components *e*_*r*_(*t*) and *e*_*s*_(*t*) can then be written as

(7) $$\begin{array}{c} e_{r}\left(t\right)=h_{r,i}\left(t\right)*i\left(t\right)\\ e_{s}\left(t\right)=h_{s,i}\left(t\right)*i\left(t\right) \end{array}$$

where *h*_*r,i*_(*t*) and *h*_*s,i*_(*t*) are the impulse responses of MRI receive coil and EMI sensing coil to the EMI signal *i*(*t*), respectively. Here, * denotes the 1D convolution.

Existing active EMI suppression methods assume there exists a transfer function between impulse responses of MRI receive coils and EMI sensing coils, such that the EMI signal component in MRI receive coil signal can be estimated using the transfer function and EMI sensing coil signals. Several methods have been recently proposed,^117^, ^118^, ^119^, ^120^, ^121^, ^122^, ^123^, ^124^, ^125^ mainly differing in the estimation method of the transfer function.

### 5.1 Analytical methods

A simple approach consists of estimating the EMI signal component in an MRI receive coil signal using spectral domain transfer function:

(8) $$e_{r}\left(t\right)=F^{-1}\left(H_{\mathrm{RS},i}\left(\omega \right)\cdot F\left(e_{s}\left(t\right)\right)\right),\;\text{where}\;H_{\mathrm{RS},i}\left(\omega \right)=\frac{F\left(h_{r,i}\left(t\right)\right)}{F\left(h_{s,i}\left(t\right)\right)}.$$

Here, *H*_*RS,i*_(ω) is the spectral domain transfer function between impulse responses *h*_*r,i*_(*t*) and *h*_*s,i*_(*t*); $F\left(\cdot \right)$ and $F^{-1}\left(\cdot \right)$ are operator and inverse operator of Fourier transform, respectively.

When multiple EMI sensing coils are available, Equation (8) can be extended as

(9) $$e_{r}\left(t\right)=F^{-1}\left(\sum_{n=1}^{N}H_{RS_{n},i}\left(\omega \right)\cdot F\left(e_{s_{n}}\left(t\right)\right)\right)$$

where $H_{RS_{n},i}\left(\omega \right)$ denotes the spectral domain transfer function between the MRI receive coil and the *n*‐th (*n* = 1, 2, …, *N*) EMI sensing coil; $e_{s_{n}}\left(t\right)$ denotes the EMI signal component in signal $y_{s_{n}}\left(t\right)$ that is detected by the *n*‐th EMI sensing coil.

With the relationship between EMI signal components *e*_*r*_(*t*) and *e*_*s*_(*t*) known, the EMI signal component *e*_*r*_(*t*) in MRI receive coil signal *y*_*r*_(*t*) in Equation (6) can then be predicted and removed from MR signal by

(10) $$y_{r}^{c}\left(t\right)=y_{r}\left(t\right)-F^{-1}\left(\sum_{n=1}^{N}H_{RS_{n},i}\left(\omega \right)\cdot F\left(e_{s_{n}}\left(t\right)\right)\right)$$

Here, $y_{r}^{c}\left(t\right)$ is the MRI receive coil signal after EMI cancellation. The abovementioned EMI cancellation approach has been demonstrated for a 64‐mT head MRI scanner,^117^, ^120^ a 80‐mT head MRI scanner^126^ with partial RF shielding, and a 50‐mT head MRI scanner.^123^ In their implementations, EMI characterization data was acquired in the absence of any MRI signal (e.g., no RF excitation)^117^, ^126^ or on data picked on the periphery of *k‐*space.^123^ Spectral domain transfer functions were then estimated by numerically fitting EMI sensing coil spectra to the MRI receive coil spectrum (e.g., via least squares minimization). In the presence of noise, this generalized approach remains largely valid even with multiple EMI sources.

More recently, this approach was extended for time domain implementation as linear convolutions with the assumption that the spectral domain transfer functions have limited spatial supports.^121^ Specifically, this approach named External Dynamic Interference Estimation and Removal (EDITER) estimates the EMI signal relationships directly using MRI data (i.e., avoiding the EMI characterization data acquisition) by assuming low correlations between the MRI and EMI signals.^121^ EDITER incorporates an adaptive procedure, dividing the MRI data into temporally correlated sub‐datasets to manage dynamically varying EMI signals caused by EMI source changes. The latter yields promising imaging results in the human brain at 47 mT when used together with conductive cloth.

The human body can also serve as an effective antenna for EMI reception.^123^, ^127^, ^128^ With EDITER, authors show that an untuned electrode (originally used for ECG monitoring) attached to the patient wrist was more effective than using a resonant solenoid coil placed near the human body.^121^ Lately, Yang et al. proposed using a “ring”‐shaped coil to wear on the subject's finger to manage body antenna effects. The ring‐coil EMI suppression was compared to that of an electrode and results showed both were able to suppress human induced EMI effectively. Authors eventually suggest using both sensors simultaneously for optimized performance.^123^

To reduce the overall cost and complexity of EMI cancellation systems, Parsa et al. recently proposed an approach that does not require external EMI sensing coil(s).^129^ This approach leverages a circularly polarized RF coil (e.g., birdcage) with two separate channels in quadrature. While one channel only senses EMI (due to its B1 orientation), the other senses both MRI and EMI signals. The two channels respectively act as an EMI and an MRI coil located at the exact same location, leading, in the case of a non‐directional EMI polarization, to a spectral domain transfer function *H*_*RS,i*_(ω) = 1. Equipped with multiple receive channel spectrometers, a simple post‐acquisition subtraction can be performed to remove EMI signals: $y_{r}^{c}\left(t\right)=y_{r}\left(t\right)-e_{s}\left(t\right)$. Further considering limitations to a single channel receive spectrometer, Parsa et al. subsequently proposed a 180° power combiner to subtract the two signals directly in hardware instead of through post‐processing.

### 5.2 Deep learning–based methods

Analytical methods assume a linear relationship between signals detected by EMI sensing coils and the primary MRI receiver coil. In practice, such a linear relationship is not always valid. For instance, if the number of EMI sensing coils is smaller than the number of EMI sources, finding the transfer function becomes an ill‐posed problem. Conversely, deep learning approaches such as non‐linear time domain convolution neural network models are expected to better approximate the transfer function in practice.^125^

**FIGURE 9:**
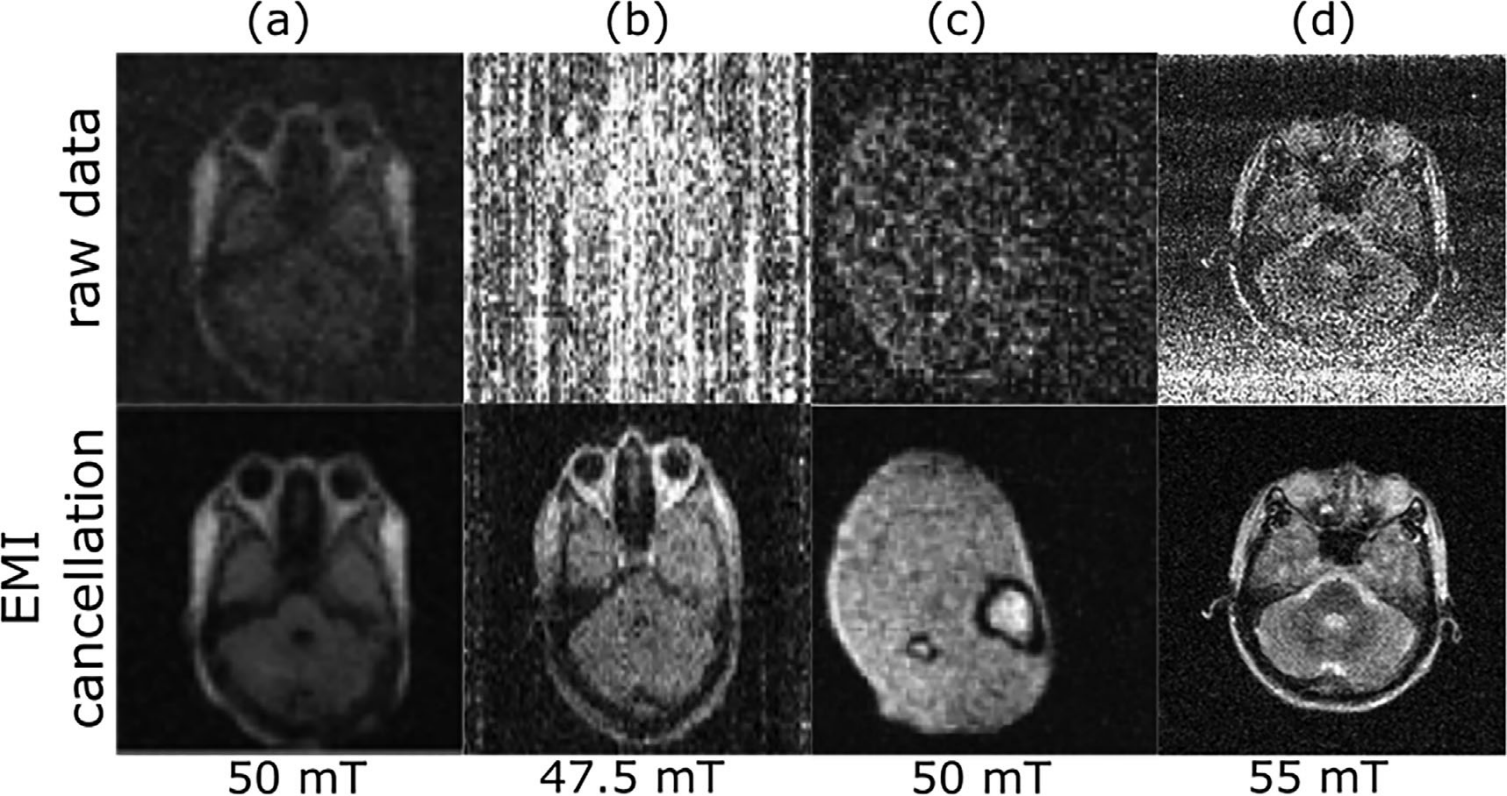
Examples of in vivo EMI cancellation of different works. (A) EMI correction of brain images acquired on a shielding‐free 50 mT scanner using an electrode and a ring‐coil.^123^ (B) EDITER correction of brain images acquired on a 47.5‐mT scanner within a one‐sided open copper box using an electrode and one pick‐up coil.^121^ (C) Single‐coil‐based EMI correction using combined ports technique (180 power splitter/combiner) of an in vivo leg images acquired on 50 mT scanner without shielding cloth and with an open Faraday shield.^129^ (D) Deep learning–based EMI correction of brain images acquired on a shielding‐free 55 mT scanner using 10 pick‐up coils placed around the scanner and inside electronic cabinet.^119^

A deep learning–based method has been developed to establish the relationships among EMI signals detected by EMI sensing coils and MRI receive coil in a data‐driven manner via a CNN model.^116^, ^119^, ^124^, ^125^ The model was trained with scan‐specific EMI characterization data acquired during the dead time of each TR, and its implementation was relatively straightforward, producing nearly complete EMI removal for a 55‐mT brain MRI scanner with no RF shielding, even in the presence of multiple external and internal EMI sources. This method was demonstrated on a 1.5‐T whole‐body MRI scanner with incomplete RF shielding.^130^ Showing high performance, this method requires a training step after each scan which increases the overall time of the experiment. For EMI signal characterization, the sequence is modified by adding a time‐overhead during each TR which may increase the shortest possible TR. Eventually, a total of 10 EMI sensors were used in this work which may also contribute to enhanced overall performance. Active efforts are currently under way for more effective deep learning approaches in the presence of strong and complex EMI signals with fewer sensors (i.e., to mitigate overall costs and complexity) and without the EMI signal characterization time overhead. Examples of in vivo EMI cancellation of different works are shown in Figure 9.

## 6 SUMMARY AND FUTURE DIRECTIONS

**FIGURE 10:**
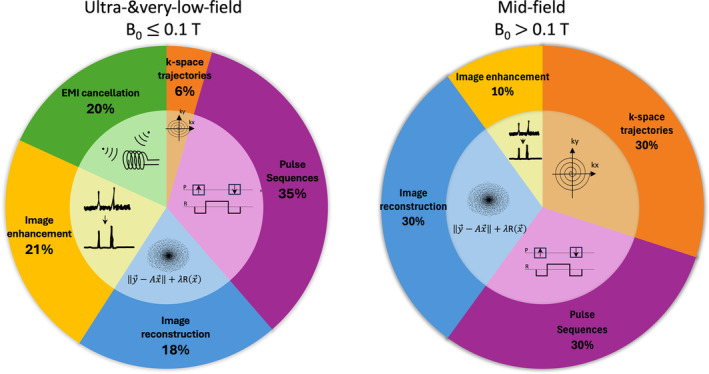
Overview of software currently adopted by different low‐field regimes.

In this work, a review of software solutions for improved SNR efficiency at low fields is presented, focusing on four different aspects: *k‐*space sampling, reconstruction strategies, image processing, and EMI cancellation. A graphical summary of current software solutions to boost SNR efficiency is presented in Figure 10, for two main categories of low‐field regime: very‐ and ultra‐low‐field ($B_{0}\leq$ 0.1 T) and mid‐field ($B_{0}>$ 0.1 T). Proportions inferred for each category are based on all cited references in the proposed review, compiled in Table S1 of Supplementary Materials. Detailed explanation of the obtained proportions is made in Supplementary Materials.

Despite being only a snapshot of a dynamic research field, this chart shows that a single one‐fits‐all strategy for boosting SNR efficiency is not always relevant for the entire low‐field range, where the environment, hardware components, and physical properties of ^1^H nuclei change.

The choice of SNR enhancement methods can be cost‐driven. Low‐field MRI is often motivated to achieve lower costs and hardware components (e.g., magnet, gradients, and RF coils) and hence is designed with lower specifications which may hinder the adoption of certain methods. In particular, *k‐*space trajectories requiring high performance gradient power electronics such as spiral and Rosette may not be suitable.

Regarding pulse sequences, the choice of parameters is not straightforward and depends on many factors. bSSFP is usually considered more convenient for lower field acquisitions because of the increased tolerance towards off‐resonance effects and reduced $T_{1}$/$T_{2}$ ratio. However, this is not the case in every setting. Point‐of‐care MRI scanners based on Halbach magnet designs have poor absolute homogeneity and stability over time, making the application of bSSFP challenging, while spin‐echo‐based sequences may remain more relevant.

Parallel imaging and compressed sensing with high acceleration ratios might not be feasible for very low SNR data, as both methods are known to be rather SNR “greedy.” Consequently, these techniques are more likely to be encountered at the upper end of the low‐field range (~ > 0.2 T). On a more general note, constrained reconstructions for undersampled low‐field data are still in their early stages, and some contraints less viable at high field could be more relevant at low field such as phase smoothness constraints.

Considering image reconstruction and processing approaches, we observe that SNR improvement methods can often be categorized in two main subgroups: analytical and deep learning methods, each with benefits and potential drawbacks. On the one hand, analytical methods prove to be straightforward to integrate and reliable (i.e., flaws are predictable since based on physical models). On the other hand, deep learning shows overall better performance when compared to analytical methods, due to its ability to handle complex relationships. Yet, the robustness (generalization across domains) and reliability of deep learning methods can also be questioned.^131^ Therefore, incorporating uncertainty estimates along with deep learning output metrics may increase the user's trust and facilitate deep learning clinical deployment.^132^ Moreover, studies including larger validation cohorts with symptomatic patients are important to boost the reliability of deep learning approaches. Indeed, most commonly used deep learning methods are still fully supervised. The main disadvantage of these is their need for adequate (i.e., matching contrast, noise level, etc.), and sufficient ground‐truth data to build a robust model. Despite the efforts to build a low‐field database (such as M4Raw), the wide range of low‐field MRI systems used in research, however, makes it impossible to provide adequate datasets for every setting. Several strategies exist to alleviate this problem. If no appropriate training data is available, networks can be trained on out‐of‐distribution data, for instance, data acquired at high field strength. However, image priors learned from the training set may diverge from those in the testing set, resulting in a domain gap issue. Artificially corrupting the training data may help in this regard by reducing the domain gap but cannot produce entirely realistic training data.

Self‐supervised training schemes, such as the one used by Zhou et al.,^35^ as well as other methods that do not require fully sampled training data, such as SSDU,^133^ zero‐shot learning,^134^ or test‐time training,^135^ are promising solutions, particularly for low‐field MR image reconstruction and may be increasingly used in the future to improve SNR efficiency in this particularly challenging imaging regime.

Focusing on EMI cancellation strategies, a range of recent studies have shown very promising results. Being a relatively young application in (low‐field) MRI, it is expected that this research field will grow and many open questions remain to be investigated. More sensors seem to consistently bring better results, which will have an impact on cost (i.e., more receiver channels, detection electronics), although one to two sensor approaches allow satisfactory performance. One future direction is the optimization of EMI cancellation algorithms or procedures to robustly deal with dynamically varying EMI in truly shielding‐free MRI. Another direction is the development of EMI sensing technology.

## 7 CONCLUSION

At present, low‐field MRI sees a renewed interest and is clearly a growing and active research field. From the proposed review, we note that software solutions can take many paths to improve the inherently low SNR per square root of time typically encountered at low field. These approaches profit not only from the legacy of past and ongoing research in commercial MRI systems but also from original, creative approaches and hardware advances such as GPU computing, opening new perspectives for innovative reconstruction algorithms. EMI cancelling illustrates another of these recent original and exciting techniques that appeared at low field. The efficiency of EMI cancelling algorithms proved highly promising and beneficial especially for the recent growing category of point‐of‐care MR scanners. Furthermore, considering the large range of low field (0–1 T), it is important to embrace that one solution certainly will not fit all. Consequently, it is essential to explore the peculiarities of the different field regimes and to look instead for tailored approaches best suited to such peculiarities. All in all, we believe these advances will continue to develop and further contribute to establish low‐field MR imaging as an exciting and sustainable imaging modality in the future.

## CONFLICT OF INTEREST STATEMENT

M. Vornehm receives part of his PhD salary from Siemens Healthineers and F. Knoll receive research funding from Siemens Healthineers.

## Supporting information


**Table S1:** Low field studies cited in this paper with the corresponding B_0_ field and the methods used to enhance SNR efficiency.

## Data Availability

Data sharing is not applicable to this article as no datasets were generated or analyzed during the current study.
