# Supplementary material for: MRI at low field: A review of software solutions for improving SNR
Source: NMR Biomed. 2024 Oct 7;38(1):e5268. doi: 10.1002/nbm.5268 (PMC11605168; doi:10.1002/nbm.5268)
Supplement: Supplementary file 1 — Table S1: Low field studies cited in this paper with the corresponding B0 field and the methods used to enhance SNR efficiency. [file NBM-38-e5268-s001.docx]

Supplementary materials:

Table 1: Low field studies cited in this paper with the corresponding B_0_ field and the methods used to enhance SNR efficiency.

| **References** | $\mathbf{B}_{\mathbf{0}}$ | **k-space sampling trajectory** | **Pulse sequences** | **Image reconstruction** | **Image enhancement** | **EMI cancellation** |
| --- | --- | --- | --- | --- | --- | --- |
| (Hsu et al., 2016a) | 50 $\mu$T | Rotary&Cartesian | Spin-echo | Constrained reconstruction | - | - |
| (Sarracanie et al., 2014a) | 6.5 mT | Cartesian | bSSFP | Compressed Sensing | - | - |
| (Sarracanie et al., 2015) | 6.5 mT | Cartesian | bSSFP | Compressed Sensing | - | - |
| (Koonjoo et al., 2021a) | 6.5 & 47 mT | Cartesian | FSE, bSSFP | - | Denoising | - |
| (Srinivas et al., 2022a) | 47.5 & 80 mT | Cartesian | FSE | - | - | YES |
| (Yang et al., 2022a) | 50 mT | Cartesian | GRE | - | - | YES |
| (Parsa et al., 2023a) | 50 mT | Cartesian | FSE | - | - | YES |
| (Shan & van Gijzen, 2022a) | 50 mT | Cartesian | Spin-echo | Constrained reconstruction | Denoising | - |
| (de Leeuw den Bouter et al., 2021) | 50 mT | Cartesian | Spin-echo | Constrained reconstruction | Denoising | - |
| (de Leeuw den Bouter et al., 2022) | 50 mT | Cartesian | FSE | - | Super resolution | - |
| (Zhang et al., 2022a) | 50 mT | Cartesian | FAST | - | Denoising | - |
| (Deoni et al., 2022) | 50 mT | Cartesian | FSE | - | Super-resolution | - |
| (Koolstra et al., 2021) | 50 mT | Cartesian | FSE | Constrained reconstruction | - | - |
| (Ahishakiye, Ahishakiye, et al., 2020) | 50 mT | Cartesian | Spin-echo | Dictionary learning | - | - |
| (Zhao, Ding, et al., 2024) | 50 mT | Cartesian, EPI | GRE, FSE, bSSFP | - | Super-resolution | YES |
| (Man et al., 2023) | 55 mT | Cartesian | FSE | - | Denoising + Super-resolution | YES |
| (Zhao et al., 2023b) | 55 mT | Cartesian | GRE, FSE | - | - | YES |
| (Lau et al., 2023a) | 55 mT | Cartesian | FSE | - | Super-resolution | YES |
| (Zhao, Xiao, et al., 2024) | 55 mT | Cartesian | GRE, FSE | - | - | YES |
| (Liu et al., 2021) | 55 mT | Cartesian&EPI | GRE+FSE | - | - | YES |
| (Iglesias et al., 2023) | 64 mT | Cartesian | GRE (FLAIR) | - | Super-resolution | - |
| (Zhou et al., 2022) | 64 mT | Spiral | Spin echo | Deep learning | - | YES |
| (Guallart-Naval et al., 2022a) | 72 mT | Cartesian | FSE | - | Denoising | - |
| (Cooley et al., 2021) | 80 mT | Cartesian | FSE | Model-based | - | - |
| (Ayde et al., 2022) | 0.1 T | Cartesian | GRE | Deep learning | - | - |
| (Simonetti & Ahmad, 2017) | 0.35 T | Cartesian | bSSFP, spoiled GRE, FSE | Compressed sensing | - | - |
| (Rosenbaum et al., 2000a) | 0.35 T | Cartesian | Spin echo | - | Denoising | - |
| (Campbell-Washburn, Ramasawmy, Restivo, et al., 2019) | 0.55 T | Spiral, Cartesian | FSE | Parallel imaging | - | - |
| (Restivo et al., 2020a) | 0.55 T | Spiral, EPI, Cartesian | bSSFP | Parallel imaging | - | - |
| (Campbell-Washburn et al., 2021a) | 0.55 T | Spiral | MRF | - | - | - |
| (Tian et al., 2023) | 0.55 T | Spiral | bSSFP | - | - | - |
| (Cummings et al., 2019) | 0.55 T | Rosette | MRF | - | - | - |
| (Liu et al., 2022a) | 0.55 T | Rosette | MRF | - | - | - |
| (Muñoz et al., 2022) | 0.55 T | Cartesian | GRE | Parallel imaging | - | - |
| (Behl, 2021) | 0.55 T | Cartesian | FSE | - | Denoising | - |
| (Varghese et al., 2023) | 0.55 T | Cartesian | bSSFP, FSE, GRE | Compressed sensing +Parallel imaging | - | - |
| (Tamada & Kose, 2014) | 1 T | Cartesian | Spin echo | Compressed sensing | - | - |

**Pie chart proportions of figure 10**

The numbers/proportions shown in the pie chart are inferred from table 1 above that compiles all cited work in the manuscript and the methods used to enhance SNR efficiency: k-space sampling trajectory, pulse sequences, image reconstruction, image enhancement and EMI cancellation.

A single study can however leverage various methods. Ultimately, we counted each method individually and the total count was normalized by the total number of methods overall (i.e., independent from the number of studies), for a given magnetic field category (mid-field on the one hand, and ultra-low and very low-field on the other hand).

Table 2 compiles SNR efficiency methods for the ultra-low and very-low field MRI category.

Table 2

| **SNR efficiency methods** | **Studies that have used a specific SNR efficiency method** | **Number of studies** | **Total** |
| --- | --- | --- | --- |
| **k-space sampling trajectory** | (Liu et al., 2021; Zhao, Ding, et al., 2024; Zhou et al., 2022) | 3 (6%) | 49 |
| **Pulse sequences** | (Cooley et al., 2021; M. L. de Leeuw den Bouter et al., 2022b; Deoni et al., 2022; Guallart-Naval et al., 2022b; Koolstra et al., 2021; Koonjoo et al., 2021b; Lau et al., 2023b; Liu et al., 2021; Man et al., 2023; Parsa et al., 2023b; Sarracanie et al., 2014b, 2015; Shan & van Gijzen, 2022b; Srinivas et al., 2022b; Zhao, Ding, et al., 2024; Zhao et al., 2023a; Zhao, Xiao, et al., 2024) | 17 (35%) |  |
| **Image reconstruction** | (Ahishakiye, Van Gijzen, et al., 2020; Ayde et al., 2022; M. de Leeuw den Bouter et al., 2021b; Hsu et al., 2016b; Koolstra et al., 2021; Sarracanie et al., 2014b, 2015; Shan & van Gijzen, 2022b; Zhou et al., 2022) | 9 (18%) |  |
| **Image enhancement** | (M. de Leeuw den Bouter et al., 2021b; M. L. de Leeuw den Bouter et al., 2022b; Deoni et al., 2022; Guallart-Naval et al., 2022b; Koonjoo et al., 2021b; Lau et al., 2023b; Man et al., 2023; Shan & van Gijzen, 2022b; Zhang et al., 2022b; Zhao, Ding, et al., 2024) | 10 (21%) |  |
| **EMI cancellation** | (Lau et al., 2023b; Liu et al., 2021; Man et al., 2023; Parsa et al., 2023b; Srinivas et al., 2022b; Yang et al., 2022b; Zhao, Ding, et al., 2024; Zhao et al., 2023a; Zhao, Xiao, et al., 2024; Zhou et al., 2022) | 10 (20%) |  |

Similarly, table 3 compiles SNR efficiency methods for the mid-field MRI category.

Table 3

| **SNR efficiency methods** | **Studies that have used a specific SNR efficiency method** | **Number of studies** | **Total** |
| --- | --- | --- | --- |
| **k-space sampling trajectory** | (Campbell-Washburn et al., 2021b; Campbell-Washburn, Ramasawmy, & Restivo, 2019; Cummings et al., 2019; Liu et al., 2022b; Restivo et al., 2020b; Tian & Nayak, 2023) | 6 (30%) | 20 |
| **Pulse sequences** | (Behl, 2021; Campbell-Washburn, Ramasawmy, & Restivo, 2019; Restivo et al., 2020b; Simonetti & Ahmad, 2017; Tian & Nayak, 2023; Varghese et al., 2023) | 6 (30%) |  |
| **Image reconstruction** | (Campbell-Washburn, Ramasawmy, & Restivo, 2019; Muñoz et al., 2022; Restivo et al., 2020b; Simonetti & Ahmad, 2017; Tamada & Kose, 2014; Varghese et al., 2023) | 6 (30%) |  |
| **Image enhancement** | (Behl, 2021; Rosenbaum et al., 2000b) | 2 (10%) |  |
| **EMI cancellation** | - | 0 (0%) |  |

**References**

Ahishakiye, E., Ahishakiye, E., Van Gijzen, M. B., Tumwiine, J., & Obungoloch, J. (2020). Adaptive-size dictionary learning using information theoretic criteria for image reconstruction from undersampled k-space data in low field magnetic resonance imaging. *BMC Medical Imaging*, *20*(1), 1–12. https://doi.org/10.1186/s12880-020-00474-3

Ahishakiye, E., Van Gijzen, M. B., Tumwiine, J., & Obungoloch, J. (2020). A Dictionary Learning Approach for Noise-Robust Image Reconstruction in Low-Field Magnetic Resonance Imaging. *2020 IST-Africa Conference, IST-Africa 2020*, 1–12.

Ayde, R., Senft, T., Salameh, N., & Sarracanie, M. (2022). Deep learning for fast low-field MRI acquisitions. *Scientific Reports*, *12*(1), 1–13. https://doi.org/10.1038/s41598-022-14039-7

Behl, N. (2021). Deep Resolve – Mobilizing the Power of Networks. *MAGNETOM Flash*, *78*, 2–9.

Campbell-Washburn, A. E., Jiang, Y., Körzdörfer, G., Nittka, M., & Griswold, M. A. (2021a). Feasibility of MR fingerprinting using a high-performance 0.55 T MRI system. *Magnetic Resonance Imaging*, *81*(June), 88–93. https://doi.org/10.1016/j.mri.2021.06.002

Campbell-Washburn, A. E., Jiang, Y., Körzdörfer, G., Nittka, M., & Griswold, M. A. (2021b). Feasibility of MR fingerprinting using a high-performance 0.55 T MRI system. *Magnetic Resonance Imaging*, *81*(June), 88–93. https://doi.org/10.1016/j.mri.2021.06.002

Campbell-Washburn, A. E., Ramasawmy, R., & Restivo, M. C. (2019). Opportunities in Interventional and Diagnostic Imaging by. *Radiology*, *3*, 2–11.

Campbell-Washburn, A. E., Ramasawmy, R., Restivo, M. C., Bhattacharya, I., Basar, B., Herzka, D. A., Hansen, M. S., Rogers, T., Patricia Bandettini, W., McGuirt, D. R., Mancini, C., Grodzki, D., Schneider, R., Majeed, W., Bhat, H., Xue, H., Moss, J., Malayeri, A. A., Jones, E. C., … Balaban, R. S. (2019). Opportunities in interventional and diagnostic imaging by using high-performance low-field-strength MRI. *Radiology*, *293*(2), 384–393. https://doi.org/10.1148/radiol.2019190452

Cooley, C. Z., McDaniel, P. C., Stockmann, J. P., Srinivas, S. A., Cauley, S. F., Śliwiak, M., Sappo, C. R., Vaughn, C. F., Guerin, B., Rosen, M. S., Lev, M. H., & Wald, L. L. (2021). A portable scanner for magnetic resonance imaging of the brain. *Nature Biomedical Engineering*, *5*(3), 229–239. https://doi.org/10.1038/s41551-020-00641-5

Cummings, E., Liu, Y., Jiang, Y., Ropella-Panagis, K., Hamilton, jesse, & Seiberlich, N. (2019). Simultaneous mapping of T1, T2, and T2* at 0.55T with Rosette MR fingerprinting. *Proceedings of the International Society for Magnetic Resonance in Medicine*, *2*(2), 1–6.

de Leeuw den Bouter, M. L., Ippolito, G., O’Reilly, T. P. A., Remis, R. F., van Gijzen, M. B., & Webb, A. G. (2022a). Deep learning-based single image super-resolution for low-field MR brain images. *Scientific Reports*, *12*(1), 1–10. https://doi.org/10.1038/s41598-022-10298-6

de Leeuw den Bouter, M. L., Ippolito, G., O’Reilly, T. P. A., Remis, R. F., van Gijzen, M. B., & Webb, A. G. (2022b). Deep learning-based single image super-resolution for low-field MR brain images. *Scientific Reports*, *12*(1), 1–10. https://doi.org/10.1038/s41598-022-10298-6

de Leeuw den Bouter, M., van Gijzen, M., & Remis, R. (2021a). Low-field magnetic resonance imaging using multiplicative regularization. *Magnetic Resonance Imaging*, *75*(September 2020), 21–33. https://doi.org/10.1016/j.mri.2020.10.001

de Leeuw den Bouter, M., van Gijzen, M., & Remis, R. (2021b). Low-field magnetic resonance imaging using multiplicative regularization. *Magnetic Resonance Imaging*, *75*(October 2020), 21–33. https://doi.org/10.1016/j.mri.2020.10.001

Deoni, S. C. L., O’Muircheartaigh, J., Ljungberg, E., Huentelman, M., & Williams, S. C. R. (2022). Simultaneous high-resolution T2-weighted imaging and quantitative T2 mapping at low magnetic field strengths using a multiple TE and multi-orientation acquisition approach. *Magnetic Resonance in Medicine*, *88*(3), 1273–1281. https://doi.org/10.1002/mrm.29273

Guallart-Naval, T., Algarín, J. M., Pellicer-Guridi, R., Galve, F., Vives-Gilabert, Y., Bosch, R., Pallás, E., González, J. M., Rigla, J. P., Martínez, P., Lloris, F. J., Borreguero, J., Marcos-Perucho, Á., Negnevitsky, V., Martí-Bonmatí, L., Ríos, A., Benlloch, J. M., & Alonso, J. (2022a). Portable magnetic resonance imaging of patients indoors, outdoors and at home. *Scientific Reports*, *12*(1), 13147. https://doi.org/10.1038/s41598-022-17472-w

Guallart-Naval, T., Algarín, J. M., Pellicer-Guridi, R., Galve, F., Vives-Gilabert, Y., Bosch, R., Pallás, E., González, J. M., Rigla, J. P., Martínez, P., Lloris, F. J., Borreguero, J., Marcos-Perucho, Á., Negnevitsky, V., Martí-Bonmatí, L., Ríos, A., Benlloch, J. M., & Alonso, J. (2022b). Portable magnetic resonance imaging of patients indoors, outdoors and at home. *Scientific Reports*, *12*(1), 13147. https://doi.org/10.1038/s41598-022-17472-w

Hsu, Y. C., Zevenhoven, K. C. J., Chu, Y. H., Dabek, J., Ilmoniemi, R. J., & Lin, F. H. (2016a). Rotary scanning acquisition in ultra-low-field MRI. *Magnetic Resonance in Medicine*, *75*(6), 2255–2264. https://doi.org/10.1002/mrm.25676

Hsu, Y. C., Zevenhoven, K. C. J., Chu, Y. H., Dabek, J., Ilmoniemi, R. J., & Lin, F. H. (2016b). Rotary scanning acquisition in ultra-low-field MRI. *Magnetic Resonance in Medicine*, *75*(6), 2255–2264. https://doi.org/10.1002/mrm.25676

Iglesias, J. E., Schleicher, R., Laguna, S., Billot, B., Schaefer, P., McKaig, B., Goldstein, J. N., Sheth, K. N., Rosen, M. S., & Kimberly, W. T. (2023). Quantitative Brain Morphometry of Portable Low-Field-Strength MRI Using Super-Resolution Machine Learning. *Radiology*, *306*(3). https://doi.org/10.1148/radiol.220522

Koolstra, K., O’Reilly, T., Börnert, P., & Webb, A. (2021). Image distortion correction for MRI in low field permanent magnet systems with strong B0 inhomogeneity and gradient field nonlinearities. *Magnetic Resonance Materials in Physics, Biology and Medicine*, *34*(4), 631–642. https://doi.org/10.1007/s10334-021-00907-2

Koonjoo, N., Zhu, B., Bagnall, G. C., Bhutto, D., & Rosen, M. S. (2021a). Boosting the signal-to-noise of low-field MRI with deep learning image reconstruction. *Scientific Reports*, *11*(1), 1–16. https://doi.org/10.1038/s41598-021-87482-7

Koonjoo, N., Zhu, B., Bagnall, G. C., Bhutto, D., & Rosen, M. S. (2021b). Boosting the signal-to-noise of low-field MRI with deep learning image reconstruction. *Scientific Reports*, *11*(1), 1–16. https://doi.org/10.1038/s41598-021-87482-7

Lau, V., Xiao, L., Zhao, Y., Su, S., Ding, Y., Man, C., Wang, X., Tsang, A., Cao, P., Lau, G. K. K., Leung, G. K. K., Leong, A. T. L., & Wu, E. X. (2023a). Pushing the limits of low-cost ultralow-field MRI by dual-acquisition deep learning 3D superresolution. *Magnetic Resonance in Medicine*, *March*, 1–17. https://doi.org/10.1002/mrm.29642

Lau, V., Xiao, L., Zhao, Y., Su, S., Ding, Y., Man, C., Wang, X., Tsang, A., Cao, P., Lau, G. K. K., Leung, G. K. K., Leong, A. T. L., & Wu, E. X. (2023b). Pushing the limits of low-cost ultralow-field MRI by dual-acquisition deep learning 3D superresolution. *Magnetic Resonance in Medicine*, *March*, 1–17. https://doi.org/10.1002/mrm.29642

Liu, Y., Hamilton, J., Jiang, Y., & Seiberlich, N. (2022a). Assessment of MRF for simultaneous T 1 and T 2 quantification and water–fat separation in the liver at 0.55 T. *Magnetic Resonance Materials in Physics, Biology and Medicine*, *0123456789*. https://doi.org/10.1007/s10334-022-01057-9

Liu, Y., Hamilton, J., Jiang, Y., & Seiberlich, N. (2022b). Assessment of MRF for simultaneous T 1 and T 2 quantification and water–fat separation in the liver at 0.55 T. *Magnetic Resonance Materials in Physics, Biology and Medicine*, *36*(3), 513–523. https://doi.org/10.1007/s10334-022-01057-9

Liu, Y., Leong, A. T. L., Zhao, Y., Xiao, L., Mak, H. K. F., Tsang, A. C. O., Lau, G. K. K., Leung, G. K. K., & Wu, E. X. (2021). A low-cost and shielding-free ultra-low-field brain MRI scanner. *Nature Communications*, *12*(1), 1–14. https://doi.org/10.1038/s41467-021-27317-1

Man, C., Lau, V., Su, S., Zhao, Y., Xiao, L., Ding, Y., Leung, G. K. K., Leong, A. T. L., & Wu, E. X. (2023). Deep learning enabled fast 3D brain MRI at 0.055 tesla. *Science Advances*, *9*(38), eadi9327. https://doi.org/10.1126/sciadv.adi9327

Muñoz, F., Lim, Y., Cui, S. X., Stark, H., & Nayak, K. S. (2022). Evaluation of a novel 8-channel RX coil for speech production MRI at 0.55 T. *Magnetic Resonance Materials in Physics, Biology and Medicine*, *0123456789*. https://doi.org/10.1007/s10334-022-01036-0

Parsa, J., O’Reilly, T., & Webb, A. (2023a). A single-coil-based method for electromagnetic interference reduction in point-of-care low field MRI systems. *Journal of Magnetic Resonance*, *346*, 107355. https://doi.org/10.1016/j.jmr.2022.107355

Parsa, J., O’Reilly, T., & Webb, A. (2023b). A single-coil-based method for electromagnetic interference reduction in point-of-care low field MRI systems. *Journal of Magnetic Resonance*, *346*, 107355. https://doi.org/10.1016/j.jmr.2022.107355

Restivo, M. C., Ramasawmy, R., Bandettini, W. P., Herzka, D. A., & Campbell-Washburn, A. E. (2020a). Efficient spiral in-out and EPI balanced steady-state free precession cine imaging using a high-performance 0.55T MRI. *Magnetic Resonance in Medicine*, *84*(5), 2364–2375. https://doi.org/10.1002/mrm.28278

Restivo, M. C., Ramasawmy, R., Bandettini, W. P., Herzka, D. A., & Campbell-Washburn, A. E. (2020b). Efficient spiral in-out and EPI balanced steady-state free precession cine imaging using a high-performance 0.55T MRI. *Magnetic Resonance in Medicine*, *84*(5), 2364–2375. https://doi.org/10.1002/mrm.28278

Rosenbaum, W. L., Atkinsa, M. S., & Sarty, G. E. (2000a). Classification and performance of denoising algorithms for low signal to noise ratio magnetic resonance images. *Image (Rochester, N.Y.)*, *3979*, 1436–1442.

Rosenbaum, W. L., Atkinsa, M. S., & Sarty, G. E. (2000b). Signal To Noise Ratio Magnetic Resonance Images. *Image (Rochester, N.Y.)*, *3979*, 1436–1442.

Sarracanie, M., Armstrong, B. D., Stockmann, J., & Rosen, M. S. (2014a). High speed 3D overhauser-enhanced MRI using combined b-SSFP and compressed sensing. *Magnetic Resonance in Medicine*, *71*(2), 735–745. https://doi.org/10.1002/mrm.24705

Sarracanie, M., Armstrong, B. D., Stockmann, J., & Rosen, M. S. (2014b). High speed 3D overhauser-enhanced MRI using combined b-SSFP and compressed sensing. *Magnetic Resonance in Medicine*, *71*(2), 735–745. https://doi.org/10.1002/mrm.24705

Sarracanie, M., Lapierre, C. D., Salameh, N., Waddington, D. E. J., Witzel, T., & Rosen, M. S. (2015). Low-Cost High-Performance MRI. *Scientific Reports*, *5*, 1–9. https://doi.org/10.1038/srep15177

Shan, X., & van Gijzen, M. B. (2022a). Deflated preconditioned Conjugate Gradient methods for noise filtering of low-field MR images. *Journal of Computational and Applied Mathematics*, *400*, 113730. https://doi.org/10.1016/j.cam.2021.113730

Shan, X., & van Gijzen, M. B. (2022b). Deflated preconditioned Conjugate Gradient methods for noise filtering of low-field MR images. *Journal of Computational and Applied Mathematics*, *400*, 113730. https://doi.org/10.1016/j.cam.2021.113730

Simonetti, O. P., & Ahmad, R. (2017). Low-Field Cardiac Magnetic Resonance Imaging: A Compelling Case for Cardiac Magnetic Resonance’s Future. *Circulation: Cardiovascular Imaging*, *10*(6), 1–7. https://doi.org/10.1161/CIRCIMAGING.117.005446

Srinivas, S. A., Cauley, S. F., Stockmann, J. P., Sappo, C. R., Vaughn, C. E., Wald, L. L., Grissom, W. A., & Cooley, C. Z. (2022a). External Dynamic InTerference Estimation and Removal (EDITER) for low field MRI. *Magnetic Resonance in Medicine*, *87*(2), 614–628. https://doi.org/10.1002/mrm.28992

Srinivas, S. A., Cauley, S. F., Stockmann, J. P., Sappo, C. R., Vaughn, C. E., Wald, L. L., Grissom, W. A., & Cooley, C. Z. (2022b). External Dynamic InTerference Estimation and Removal (EDITER) for low field MRI. *Magnetic Resonance in Medicine*, *87*(2), 614–628. https://doi.org/10.1002/mrm.28992

Tamada, D., & Kose, K. (2014). Two-dimensional compressed sensing using the cross-sampling approach for low-field MRI systems. *IEEE Transactions on Medical Imaging*, *33*(9), 1905–1912. https://doi.org/10.1109/TMI.2014.2326864

Tian, Y., Cui, S. X., Lim, Y., Lee, N. G., Zhao, Z., & Nayak, K. S. (2023). Contrast-optimal simultaneous multi-slice bSSFP cine cardiac imaging at 0.55 T. *Magnetic Resonance in Medicine*, *89*(2), 746–755. https://doi.org/10.1002/mrm.29472

Tian, Y., & Nayak, K. S. (2023). New clinical opportunities of low-field MRI: heart, lung, body, and musculoskeletal. *Magnetic Resonance Materials in Physics, Biology and Medicine*, *0123456789*. https://doi.org/10.1007/s10334-023-01123-w

Varghese, J., Jin, N., Giese, D., Chen, C., Liu, Y., Pan, Y., Nair, N., Shalaan, M. T., Khan, M., Tong, M. S., Ahmad, R., Han, Y., & Simonetti, O. P. (2023). Building a comprehensive cardiovascular magnetic resonance exam on a commercial 0.55 T system : A pictorial essay on potential applications. *Frontiers in Cardiovascular Medicine*. https://doi.org/10.3389/fcvm.2023.1120982

Yang, L., He, W., He, Y., Wu, J., Shen, S., & Xu, Z. (2022a). Active EMI Suppression System for a 50 mT Unshielded Portable MRI Scanner. *IEEE Transactions on Biomedical Engineering*, *9294*(c). https://doi.org/10.1109/TBME.2022.3170450

Yang, L., He, W., He, Y., Wu, J., Shen, S., & Xu, Z. (2022b). Active EMI Suppression System for a 50 mT Unshielded Portable MRI Scanner. *IEEE Transactions on Biomedical Engineering*, *69*(11), 3415–3426. https://doi.org/10.1109/TBME.2022.3170450

Zhang, Y., He, W., Chen, F., Wu, J., He, Y., & Xu, Z. (2022a). Denoise Ultra-Low-Field 3D Magnetic Resonance Images Using a Joint Signal-Image Domain Filter. *Journal of Magnetic Resonance*, 107319. https://doi.org/10.1016/j.jmr.2022.107319

Zhang, Y., He, W., Chen, F., Wu, J., He, Y., & Xu, Z. (2022b). Denoise ultra-low-field 3D magnetic resonance images using a joint signal-image domain filter. *Journal of Magnetic Resonance*, *344*, 107319. https://doi.org/10.1016/j.jmr.2022.107319

Zhao, Y., Ding, Y., Lau, V., Man, C., Su, S., Xiao, L., Leong, A. T. L., & Wu, E. X. (2024). Whole-body magnetic resonance imaging at 0.05 Tesla. *Science (New York, N.Y.)*, *384*(6696), eadm7168. https://doi.org/10.1126/science.adm7168

Zhao, Y., Xiao, L., Hu, J., & Wu, E. X. (2024). Robust EMI elimination for RF shielding-free MRI through deep learning direct MR signal prediction. *Magnetic Resonance in Medicine*, *January*, 112–127. https://doi.org/10.1002/mrm.30046

Zhao, Y., Xiao, L., Liu, Y., Leong, A. T., & Wu, E. X. (2023a). Electromagnetic interference elimination via active sensing and deep learning prediction for radiofrequency shielding-free MRI. *NMR in Biomedicine*, *April*, 1–13. https://doi.org/10.1002/nbm.4956

Zhao, Y., Xiao, L., Liu, Y., Leong, A. T., & Wu, E. X. (2023b). Electromagnetic Interference (EMI) Elimination via Active Sensing and Deep Learning Prediction for RF Shielding-free MRI. *NMR in Biomedicine*, 0–1. https://doi.org/10.1002/nbm.4956

Zhou, B., Schlemper, J., Dey, N., Mohseni Salehi, S. S., Sheth, K., Liu, C., Duncan, J. S., & Sofka, M. (2022). Dual-domain self-supervised learning for accelerated non-Cartesian MRI reconstruction. *Medical Image Analysis*, *81*(October 2021), 102538. https://doi.org/10.1016/j.media.2022.102538
